# Bone Regeneration Enhanced by Quercetin-Capped Selenium Nanoparticles via miR206/Connexin43, WNT, and BMP signaling pathways

**DOI:** 10.14336/AD.2025.0025

**Published:** 2026-02-28

**Authors:** Garima Sharma, Yeon Hee Lee, Jin-Chul Kim, Ashish Ranjan Sharma, Sang-Soo Lee

**Affiliations:** ^1^Department of Biomedical Science & Institute of Bioscience and Biotechnology, Kangwon National University, Chuncheon 24341, Korea.; ^2^Institute for Skeletal Aging & Orthopedic Surgery, Hallym University-Chuncheon Sacred Heart Hospital, Chuncheon-si, 24252, Gangwon-do, Korea.

**Keywords:** Quercetin, nanoparticles, bone regeneration, bone signaling, miRNA

## Abstract

Age-related alterations in the skeletal system are linked to decreased bone mass, a reduction in bone strength and density, and an increased risk of fractures and osteoporosis. Therapeutics are desired to stimulate bone regeneration and restore imbalance in the bone remodeling process. Quercetin (Qu), a naturally occurring flavonoid, induces osteogenesis; however, its solubility, stability, and bioavailability limit its therapeutic use. Nanoformulation can improve the physical properties of Qu and enhance its bioactivity and bioavailability. Further, localized delivery of Qu nanoformulations at the site of bone defects could ensure high local concentration, augmenting its osteogenic properties. Thus, this study aims to synthesize selenium nanoparticles-based Qu nanoformulation (Qu-SeNPs) and evaluate their osteogenic stimulation ability along with localized bone regeneration ability. Here, the spontaneously synthesized Qu-SeNPs showed uniform size distribution and rough flower-shaped morphology. The confocal images indicate improved cellular uptake and even cellular distribution of Qu-SeNPs in osteoblasts, resulting in increased osteogenic activity as indicated by enhanced expression of early and late osteoprogenitor differentiation markers. Qu-SeNPs also decreased osteoblasts' RANKL/OPG ratio and inhibited osteoclast formation. Mechanistically, Qu-SeNPs activate critical signaling pathways, including WNT and BMP, and utilize the miR-206/Connexin43 pathway to enhance osteogenesis. In vivo, experiments utilizing a drill-hole bone defect model in mice indicate that hydrogel-mediated localized delivery of Qu-SeNPs significantly accelerates bone defect healing. Thus, well-characterized and mechanistic, detailed synthesized Qu-SeNPs can restore bone remodeling, and Qu-SeNPs embedded in hydrogels may improve Qu cellular uptake and bioavailability in clinical settings, enabling innovative orthopedic and regenerative therapies for bone loss/defects.

## INTRODUCTION

Skeletal integrity declines, and mechanical properties are compromised due to age-related changes in bone structure, including microarchitectural alterations and a decrease in bone mass.

The reduction in bone mineral density (BMD) is a characteristic of skeletal aging. The reduction in bone strength that occurs as age increases is further exacerbated by changes in bone microarchitecture, such as increased cortical porosity and trabecular thinning. Bone remodeling is the physiological dynamic process involving a wide range of cellular functions, including proliferation, differentiation, migration, angiogenesis, and remodeling, all working in tandem in a dynamic equilibrium [1, 2]. Even bone loss resulting from trauma, surgical procedures, and tumor excision often leads to critical-sized bone defects that need filling with substitute materials. Stimulating bone regeneration and repair is highly desirable for treating bone defects by enhancing osteoblast production and inhibiting osteoclast formation, which is responsible for bone production and resorption [3]. The self-healing capacity of bone is limited and often needs external intervention to enhance repair and regeneration. Osteoporotic fractures often need pharmacological intervention to restore the balance of bone remodeling and facilitate bone regeneration and repair. Among such interventions, localized therapeutics at the site of bone injury increase local drug availability and efficacy while mitigating adverse effects related to off-target accumulation. These therapeutics can be used during the initial stage of bone regeneration, most probably during surgical procedures, where drugs can be injected or implanted directly at the site of action [4]. Although significant improvements have been made in bone defect management, developing new treatment approaches that can stimulate bone regeneration and repair in debilitating conditions is still a requisite in biomedical research.

Quercetin (3,3′,4′,5,7-pentahydroxyflavone, Qu) is a scientifically proven natural flavonoid with promising potential in the therapeutics [5]. The Qu is known to elevate alkaline phosphatase (ALP) levels and improve bone mineralization in osteoblast-like mouse calvaria (MC3T3-E1) cell line [6] and can suppress the development of osteoclasts by downregulating nuclear factor kappa B (NF-κB) and activating protein 1 (AP-1) in femoral organ cultures [7]. It has also been reported that Qu can potentiate the WNT/β-catenin pathway [8] and upregulate the expression of BMP-2 to promote osteogenic differentiation of bone marrow mesenchymal stem cells [9]. Qu has also been shown to protect against bone loss in ovariectomized mice [10, 11]. However, the application of Qu in clinics is limited owing to its suboptimal physical properties, such as its low solubility and cellular availability [12]. Thus, a larger quantity of Qu might be necessary to achieve the desired osteogenic effect. Even though applying Qu at the site of bone defect could provide high local concentration, maximizing its osteogenic effect and reducing the risks associated with systemic administration of large quantities of Qu [13], the poor solubility and stability of Qu in an aqueous environment remains a challenge.

Since nanotechnology can offer manipulations of materials at the nanoscale level that could enhance the cellular uptake of hydrophobic compounds, such as Qu, and can promote their bone regenerating abilities, it is one of the many impending methods to develop highly potent treatments to use hydrophobic natural compounds for bone regeneration and repair at lesser quantities. In addition, NPs can also act as cutting-edge delivery methods with various advantages over more conventional drug delivery methods, such as improved delivery of hydrophobic drugs and boosted cellular uptake by target cells [14, 15]. Among various NPs, surface-functionalized selenium nanoparticles (SeNPs) have been reported as nanosized delivery tools for biomolecules [16]. Selenium (Se) and its metabolites are essential micronutrients crucial to bone health as they prevent bone resorption, among other benefits, such as antioxidant behavior, anti-inflammatory effects, and immunity functions [17, 18]. Our group previously reported that sodium selenite promotes osteoblast differentiation by regulating the Wnt/β-catenin pathway *in vitro* [19]. However, Se can show toxic effects at higher concentrations and thus is not always suitable in clinics. Thus, SeNPs could be the center of interest because of their high stability in colloidal form, augmented antioxidation property, biocompatibility, and possible medicinal uses in bone tissue engineering due to low toxicity compared to inorganic and inorganic Se [20]. These properties of SeNPs are due to their high surface area-to-volume ratio, which offers more reactive sites for biological processes and attachment sites for biomolecules. Although SeNPs are less toxic than Se, despite some uncertainties, *in vivo*, studies have demonstrated the concentration-dependent adverse effect of SeNPs, as evidenced by several indicators of general toxic action [21], which could be possibly due to the chemical surface coating of SeNPs, which play a significant role in the toxicity profile of SeNPs [22]. Therefore, replacing chemical capping agents in SeNPs with biomolecules could provide a non-toxic approach for improved cellular uptake of biomolecules.

Further, it has been reported that flavonoids can be adsorbed onto the metal surface by interacting with carbonyl groups or electrons [1]. Therefore, Qu can act as a reducing and capping agent for SeNPs. Moreover, when attached to SeNPs, the cellular uptake of Qu could be enhanced, minimizing the drawbacks associated with free Qu. Thus, this study aimed to synthesize Qu-SeNPs and evaluate their effectiveness in stimulating osteoblasts' osteogenic ability, inhibiting osteoclast formation, and promoting bone regeneration in a drill-hole bone defect mice model that simulates a critical-sized bone lesion and provides a controlled environment. In addition, the mechanism of action that underlies the osteogenic effects of Qu-SeNPs in regulating signaling pathways responsible for controlling osteoblast development and bone formation was also evaluated.

## MATERIALS AND METHODS

### Chemicals

Sodium selenite, Quercetin (Qu), Ascorbic acid, Cell counting kit-8 (CCK-8; GLPBIO), Dimethyl sulfoxide (DMSO), 4 % Paraformaldehyde, Alizarin Red S solution, Cetylpyridinium chloride, Sirius Red dye, Bouin's fluid, and Sodium hydroxide were purchased from Sigma-Aldrich, USA. Fetal Bovine Serum (FBS), Penicillin-Streptomycin Solution (P/S), and DMEM were purchased from Gibco, USA. Phosphate-Buffered Saline (PBS), RIPA buffer was purchased from T&I, Korea. The protein assay kit was purchased from Bio-Rad, USA. Cytotoxicity detection kit and TRACP and ALP double-stain kit were purchased from Takara Bio Inc., Japan. CSPD substrate was purchased from Roche, Germany. Trizol reagent, SuperScript ІІ Reverse Transcriptase, and Renilla luciferase thymidine kinase construct was purchased from Invitrogen, USA. SYBR green qPCR MasterMix was purchased from Bioneer, Korea. PVDF membrane and chemiluminescence reagents were purchased from Millipore Corporation, USA. Genefectine reagent was purchased from Genetrone, Korea. Axin-2 and BRE reporter construct was purchased from Addgene, USA. The dual-luciferase assay kit was purchased from Promega, USA.

### Synthesis of Qu-SeNPs

To synthesize Qu-SeNPs, 1 mL of 50 mM Qu in acetone was gradually added to 5 mM sodium selenite solution in deionized water with constant stirring. Then, slowly add 1 mL of ascorbic acid solution (400 mM) dropwise as the reducing agent. The reaction solution was stirred at 40-50°C for 12 h. The appearance of orange to brick-red color indicates Qu-SeNPs formations. Control SeNPs were also synthesized similarly without Qu. After completion of the reaction, the solution was centrifuged at 15,000 rpm for 30 min. The purified Qu-SeNPs or control SeNPs were collected as pellets, washed thrice with D/W, freeze-dried, and stored at 4°C till further use.

### Characterization of Qu-SeNPs

The UV-vis optical spectra of Qu-SeNPs were recorded using a UV-vis spectrophotometer (Jenway-7315 spectrophotometer) at 230 to 550 nm range and were compared with the characteristic absorption peaks of control SeNPs and Qu. Further, the hydrodynamic size, size distribution, polydispersity index (PDI), and zeta potential of Qu-SeNPs were recorded using a dynamic light scattering instrument (DLS; Otsuka (ELSZ-2000)). The morphology of the Qu-SeNPs and control SeNPs were observed using transmission electron microscopy (TEM; JEOL (JEM-2100F)) using carbon-coated grids and scanning electron microscopy (SEM; JEOL (JSM-7900F)) and conductive adhesive tape. The size and morphology of nanoparticles are crucial parametres as it can affect the biological activity, cellular uptake, and stability of Qu-SeNPs

An attenuated Fourier transform infrared spectroscopy (FTIR: PerkinElmer (Frontier)) at 500-4000 cm^-1^ wavenumber range was used to observe the surface functional groups and chemical interactions of as-prepared Qu-SeNPs by comparing it with the characteristic peaks corresponding to QU and control SeNPs. The Qu-SeNPs were further analyzed for their amorphous or crystalline structure using X-ray diffraction (XRD) patterns (Philips X’Pert-MPD diffractometer). The X-ray photoelectron spectroscopy (XPS; Thermo Fisher Scientific K Alpha+ spectrometer, Waltham, MA, USA) was used to investigate the elemental composition, binding energies, and chemical states of Se in the Qu-SeNPs. The Survey scans were collected between 1200 to 0 eV binding energy, at 150 eV pass energy and 1 eV intervals. A narrow scan of Se3d was also done at 50 eV pass energy and 0.1 eV intervals. The amount of Se and Qu in the synthesized Qu-SeNPs was estimated using ICP (Agilent-5900, Agilent Technologies Inc., CA, USA) and UV-vis spectrophotometer (JEOL (JSM-7900F)), respectively. In brief, the Qu-SeNPs were synthesized as mentioned above and centrifuged at 12,000 rpm for 30 min to separate unreacted Se ions as supernatant. The supernatant was used to quantify unreacted Se ions in inductively coupled plasma optical emission spectrum (ICP-OES) and the percent reduction of Se into SeNPs was calculated by subtracting the Se ions in the supernatant with the initial input of Se ions. Further, to determine the amount of Qu in Qu-SeNPs, the NPs were dissolved in ethanol (0.1 mg/mL) and were analyzed with a UV-vis spectrophotometer at 380 nm. The amount of Qu in the samples was quantified using a standard curve prepared by the known amount of Qu.

### Time-dependent Release of Qu from Qu-SeNPs

In brief, Qu-SeNPs were added to 0.1% polyethylene glycol (prepared in 1X PBS) at 1 mg mL^-1^ concentration. The solution was then incubated at 37 °C with stirring. The samples were withdrawn at pre-determined time points, centrifuged at 12,000 rpm for 15 min, and analyzed for Qu amount using UV-vis spectroscopy at 380 nm using a standard curve.

### Cell culture and cell viability assay

Mice preosteoblast cell line, MC3T3-E1 cells (ATCC, CRL-2593), and murine macrophage RAW 264.7 cells (ATCC, TIB-71) were cultured and maintained in 10 % FBS supplemented DMEM medium at 37 °C and 5 % CO_2_. The cytotoxic effect of control SeNPs, free Qu, and Qu-SeNPs on MC3T3-E1 cells was analyzed using CCK-8 assay for 72 h. The cells (2×10^4^ cells/well) were seeded in 96-well plates and treated with various concentrations of SeNPs, free Qu, and Qu-SeNPs, i.e., 0, 0.12, 0.25, 0.5, 1, and 2 µg mL^-1^, for 24 h. After incubation, the CCK-8 reagent was added to the wells, and the optical density was measured at a wavelength of 450 nm using a UV-Vis spectrophotometer.

### Alkaline Phosphatase (ALP) Activity

As a well-known indicator of osteoblast differentiation, ALP activity was analyzed by seeding 3 × 10^5^ MC3T3-E1 cells/well in 48-well plates. Then, SeNPs (0, 25, 50, 75, and 100 ng mL^-1^), free Qu (0, 0.12, 0.25, 0.5, 1, and 2 µg mL^-1^), and Qu-SeNPs (0, 25, 50, 75, and 100 ng mL^-1^ were treated at various concentrations for 48 h, followed by washing and addition of 100 µl of cold RIPA buffer to each well to collect cell lysate after centrifuging at 14,000 rpm for 20 min at 4 °. Later, CSPD substrate was added to 20 µl of supernatant (at 5:1 ratio) and incubated for 30 min at RT, and the intensity of luminescence was evaluated by a luminometer (Glomax, Promega, USA). The protein concentration was normalized in the total cell lysate by using the protein assay kit (Bio-Rad, MA, USA)

### Cellular uptake analysis

The time-dependent Qu uptake was analyzed as previously reported [23]. To evaluate the SeNPs-mediated cellular uptake of Qu, 3 × 10^5^ MC3T3 cells were seeded and cultured in a 48-well plate for 24 h. The cells were then treated with free Qu or Qu-SeNPs containing equivalent Qu concentration (i.e., 1 µg mL^-1^) for 6 h, 12 h, 24 h, 48 h, and 72 h. After pre-determined time points, cells were washed twice with PBS and lysed using RIPA buffer. The cell lysate was centrifuged at 12,000 rpm for 15 min at 4 °C. The pellet was collected and extracted with ethanol by vortexing to solubilize the Qu in ethanol fraction, and absorption spectra were recorded at 380 nm using a UV-vis spectrophotometer. The amount of Qu was calculated using a standard curve, and the protein concentration of total cell lysate was normalized using a protein assay kit.

### Confocal microscopic analysis

For confocal imaging, the cells were incubated for 12 h with free Qu or Qu-SeNPs at an equivalent Qu concentration (1 µg mL^-1^), washed twice after incubation, fixed with 4% PFA for 15 min, and stained with DAPI. The confocal images were taken at excitation 488 nm using a laser confocal microscope (Carl Zeiss (LSM880 with Airyscan)).

### RNA isolation and real-time RT-PCR

The MC3T3-E1 cells (5 × 10^4^ cells/well) were cultured in 24-well plates and treated with 75 ng mL^-1^ of Qu-SeNPs for 48 h. After treatment, Trizol reagent was used to collect the total RNA. miRNAs from the cells were isolated using the MiPure Cell/Tissue miRNA Kit from Vazyme (Nanjing, China). The quality and integrity of mRNAs and miRNs were determined using nanodrop (Thermo Fisher Scientific, MA, USA). The cDNA was synthesized, and RT-PCR was performed using kits (N711-03, Discover-sc WTA Kit V2 for mRNAs and (MR201-02, miRNA 1st Strand cDNA Synthesis Kit for miRNAs) from Vazyme, as per the instruction manual. The relative expression of mRNAs was standardized to GAPDH and miRNAs to U6. All results of mRNA expression were quantified by the double delta CT (ΔΔCT) method. The primer sequences are given in Supplementary Table 1.

### Protein extraction and western blotting

In brief, 5 × 10^4^ MC3T3-E1 cells/well were cultured in 24-well plates and were treated with 1 µg mL^-1^ of Qu or 75 ng mL^-1^ of Qu-SeNPs for 48 h. The cells were harvested with a solution containing RIPA buffer, phosphatase inhibitors, and protease inhibitors. The protein assay kit was used to normalize protein concentration, and SDS-polyacrylamide gel electrophoresis was performed. The protein bands were then transferred to a PVDF membrane, blocked with 5 % skim milk, and incubated overnight with antibodies against β-catenin (Cat. No. 610153, BD Biosciences), p-smad (Cat. No. 9511S, Cell signaling Technology), Connexin43 (Cx43) (Cat. No. 3512S, Cell signaling Technology), and β-actin (Cat. No. sc-47778, Santa Cruz Biotechnology) at 4 °C. The membranes were then washed with 1X TBST (Tris-buffered saline, 0.1 % Tween 20) and incubated with horseradish peroxidase-conjugated secondary antibodies (Jackson Immunoresearch, USA) at RT for 60 min. Chemiluminescence reagents were used to visualize target protein bands. β-actin was used as a loading control. Band intensities were quantified using Image J software (NIH, USA).

### Luciferase reporter assay and transfection

MC3T3-E1 cells were treated with 1 µg mL^-1^ of Qu or 75 ng mL^-1^ of Qu-SeNPs for 48 h after transfecting reporter constructs using Genefectine reagent in 48 well plates per the manufacturer’s instructions. The 1 μg construct of Axin-2, BRE, and the same amount of Renilla luciferase thymidine kinase construct was used to estimate the luciferase activity. Luciferase activities were evaluated by a luminometer. Identified reporter activity from each sample was normalized with Renilla luciferase activity (Promega Corp, USA).

For Cx43 studies, the wildtype or mutated sequences of 3’-UTR of Cx43 were cloned into pGL3 luciferase reporter vectors (pGL3-Mouse_Cx43_ 3'UTR_WT and pGL3-Mouse_Cx43_3'UTR_MUT) (Vector Builder, Korea) (Supplementary Fig. 1). Plasmids were transfected to MC3T3 E-1 cells using the transfection reagent LipofectamineTM 3000 (Invitrogen). Cx43 siRNA (mouse: 5'-GCCTTTCGCTGTAACACT CAA-3') and Scrambled Cx43 RNA: (mouse: 5'-GC TGTCAAC CTCTAACCTAGT-3') were transfected (Lipofectamine 3000) to the MC3T3 E-1 cell for studying the silencing effect of Cx43 gene on osteogenesis. 100nM of miR-206 mimics (5'-UGGAAUGUAAGGAAGUG UGUGG-3'), miR-206 inhibitor (5'-CCACACACUUCC UUACAUU CCA-3'), and miR-206 negative control NC (5’-UUCU CCGAACGUGUCACGUTT-3’) were trans-fected to MC3T3 E-1 cells before treatment with Qu-SeNPs using Lipofectamine 3000.

### Sirius Red S and Alizarin Red S staining

Alizarin Red S and Sirius Red staining was done to determine the effect of Qu-SeNPs on collagen synthesis and mineralized matrix deposition, respectively. After treating MC3T3-E1 cells with various concentrations (0, 25, 50, and 75 ng mL^-1^) of Qu-SeNPs for 7 days (within intermittent media change every second day), the fixed cells (4% paraformaldehyde for 20 min) were stained with 40 mM Alizarin Red S solution for 30 min and were observed under a microscope (Carl Zeiss, Germany). Further, to quantify mineralization, the stained cells were dissolved using 10% cetylpyridinium chloride for 1 h, and the absorbance at 570 nm was measured with a spectrophotometer. For collagen staining, the fixed cells (Bouin's fluid for 1 hour) were stained with 1 mg/ml Sirius Red dye dissolved in saturated aqueous picric acid for 1 h. The stained cells were further quantified by dissolving in 0.1 N NaOH for 30 min, and the optical density was measured using a spectrophotometer at 550 nm.

### Tartrate-resistant acid phosphatase (TRAP) staining

To determine the effect of Qu-SeNPs in the formation of osteoclast-like cells, 5 × 10^4^ cells RAW 264.7 cells/well were treated with RANKL 50 μg mL^-1^ for 72 h. Post-incubation, the cells were washed with PBS and treated with 75 ng mL^-1^ of Qu-SeNPs for 7 d. Cells were fixed and stained using a TRACP and ALP double-stain kit per the manufacturer's protocol. TRAP-positive multi-nucleated cells with over three nuclei were considered osteoclast-like cells and were counted under a light microscope.

### Drill hole bone defect mice model

Male ICR mice were used for surgery (10 weeks old and weighed between 26 and 28 gms). The Animal Experimentation Ethics Committee at Hallym University approved any experimental activities conducted with animals (HallymR1 (2020-37). The drill-hole bone defect model was generated in mice with slight alterations [24]. In brief, anesthesia was given to mice, and a skin incision was performed across the medial proximal end of the tibia. After removing soft tissue, a 23-gauge needle was used to drill a 0.6-mm hole in the tibia. A hole was drilled through the total diameter of the tibia, namely at the distal end of the tibial crest, passing through both the medial and lateral cortices and the intervening medulla. The holes were flushed with saline solution by injection to eliminate bone pieces. For treatment, the Qu-SeNPs or free Qu were added in a hydrogel (VitroGel Hydrogel Matrix, The Well Bioscience, New Jersey, USA) at a concentration 100 times higher than effect concentrations *in vitro*, as reported by others for localized delivery of flavonoids in mice bone defect repair [25]. Following surgery, the mice were divided into 4 groups (n = 5 in each group): control group (without any treatment), hydrogel group (treated with only hydrogel), hydrogel + Qu group (treated with hydrogel embedded with free Qu), and hydrogel + Qu-SeNPs group (treated with hydrogel embedded with Qu-SeNPs). The holes were flooded with 5 µL of the treatment samples. The skin was sutured after the treatment. Subsequently, the same surgery was performed on the contralateral limb. The mice were assessed daily for any atypical behavior, weight reduction, or decreased food consumption, although no such occurrences were detected. After 7 days of surgery, mice were euthanized, and tibae were collected for further analysis.

### microCT Analysis

The distal tibia and tibial shaft 2 mm in length covering the drill-hole site were scanned with Quantum GX μCT imaging system (PerkinElmer, Hopkinton, MA, USA) at Korea Basic Science Institute (Gwangju, Korea) with a voltage of 70 keV and current of 114 μA. To avoid sampling errors by random deviations, sections were evaluated to complete secondary spongiosa of the distal tibia. After automatic identification of the outer contour of the bone, 2D slices (19 μm isotropic resolution) were generated, and the region of interest (ROI) was selected to reconstruct the 3D image. Two ROIs were selected for the initial analysis, including posterior and anterior cortical defects, and another ROI was located in the intramedullary space between the defects.

**Figure 1.**
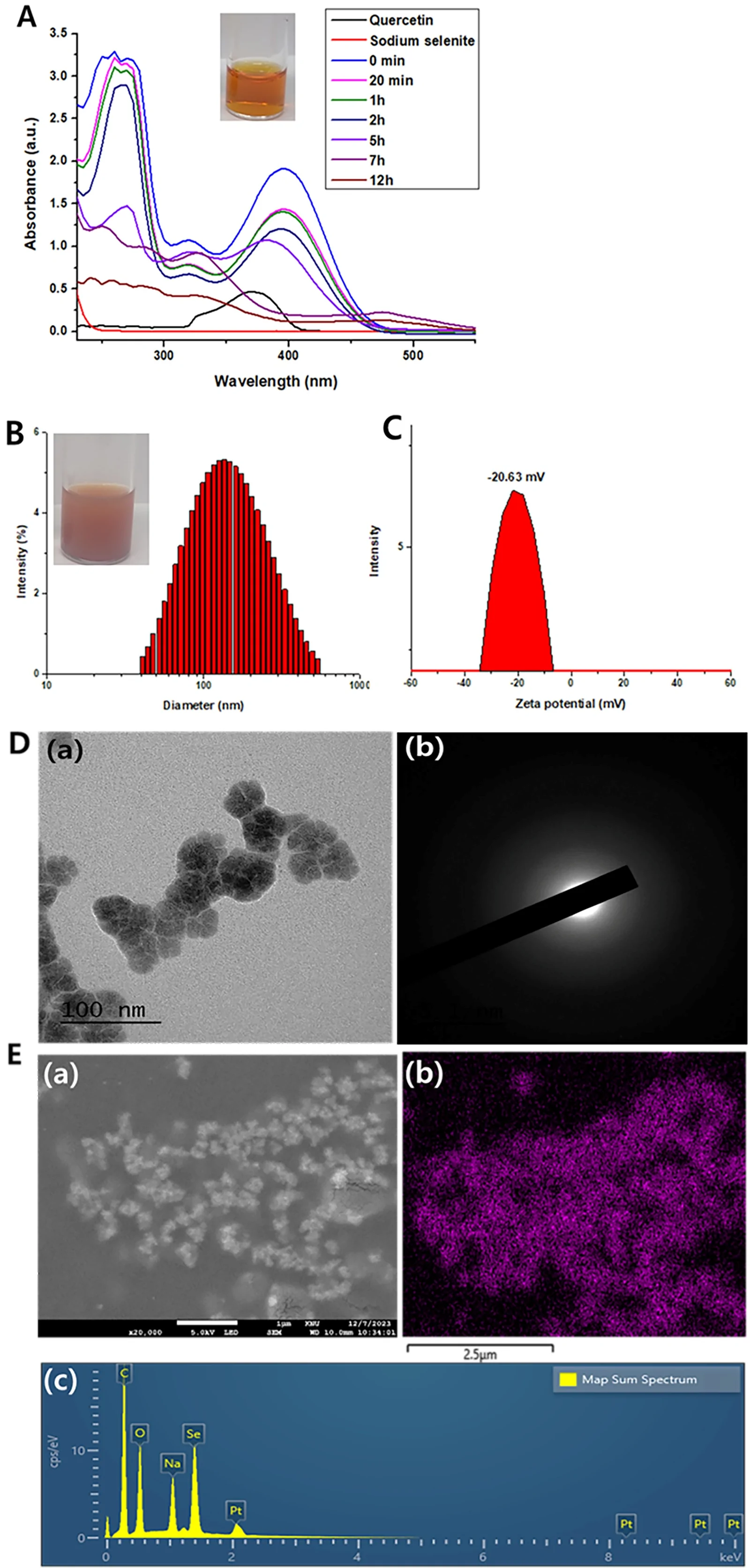
**Size and morphological characterization of Qu-SeNPs**. (**A**) UV-vis absorption spectra of Qu, sodium selenite, and time-dependent synthesis of Qu-SeNPs (inset showing the digital image of Qu-SeNPs after synthesis). (**B**) Hydrodynamic size (inset showing the digital image of centrifuged and re-disperesed Qu-SeNPs), (C) Zeta potential, (D) TEM (a) and SAED (b) images, (E) SEM image (a) and EDX analysis (b & c) of Qu-SeNPs.

The bone volume fraction (BV/TV), trabecular number (Tb.N), trabecular separation (Tb. Sp), connectivity density (Conn.D), and bone mineral density (BMD) were calculated using built-in software.

### Statistical analysis

Data normality was assessed using the Shapiro-Wilk test. Additionally, a normal Q-Q plot was generated to visually inspect the distribution of residuals. The data is represented as mean ± SD. To analyze the data statistically, Graphpad Prism 8.0 (San Diego, CA) software was used. Data were evaluated by the two-tailed Student’s t-test, one-way or two-way ANOVA, and a value of P<0.05 was considered statistically significant.

## RESULTS

### Characterization of Qu-SeNPs

The presence of SeNPs in the reaction solutions was confirmed after 24 h of reaction, as evidenced by the appearance of the distinctive orange color of SeNPs [26], indicating that the tetravalent selenium is reduced to amorphous SeNPs [27]. UV-vis absorbance of Qu-SeNPs was recorded because Qu itself has strong UV-Vis absorption in the 250-400 nm range due to its flavonoid structure and a shift in the absorption peak after capping indicates successful binding of quercetin to selenium nanoparticles. As shown in Figure 1A, at 0 min, the reaction solution of Qu-SeNPs synthesis exhibited two distinct UV-vis absorbance peaks at 265 nm and 393 nm. Given that the pristine Qu exhibited an absorbance peak at 370 nm, it can be inferred that the observed peak at 393 nm in the Qu-SeNPs reaction solution is due to the presence of the Qu, while absorbance at 265 is well-known for ascorbic acid. Further, with increased time, the intensities of both peaks were reduced, representing the consumption of ascorbic acid during the reduction of SeO_3_^2-^ to Se^0^; and the alterations in the structure of the Qu due to the non-covalent interactions of Qu molecules with SeNPs. The as-synthesized Qu-SeNPs showed a broad region of high UV-vis absorbance from 230 to 400 nm and did not form a sharp absorbance maximum peak, possibly due to the non-metal nature of Se, confirming the synthesis of SeNPs [28]. A similar time-dependent UV-vis absorbance pattern was also observed in control SeNP synthesis (Supplementary Fig. 2).

In addition, the UV-vis absorption spectral feature of SeNPs and the color of SeNPs colloidal solution are considered as a function of their size because they belong to the family of elemental semiconductors [29]. Since the smaller SeNPs show a yellow color and larger size SeNPs show a red color [29], in this study, the orange color of the reaction solution indicates the formation of small-sized Qu-SeNPs. After 12 h of reaction, the ICP analysis confirmed around 95% and 97% reduction of Se ions in the supernatant of Qu-SeNPs and control SeNPs, respectively. The amount of Qu in Qu-SeNPs was calculated to be 46.8% of the total weight of Qu-SeNPs.

**Figure 2.**
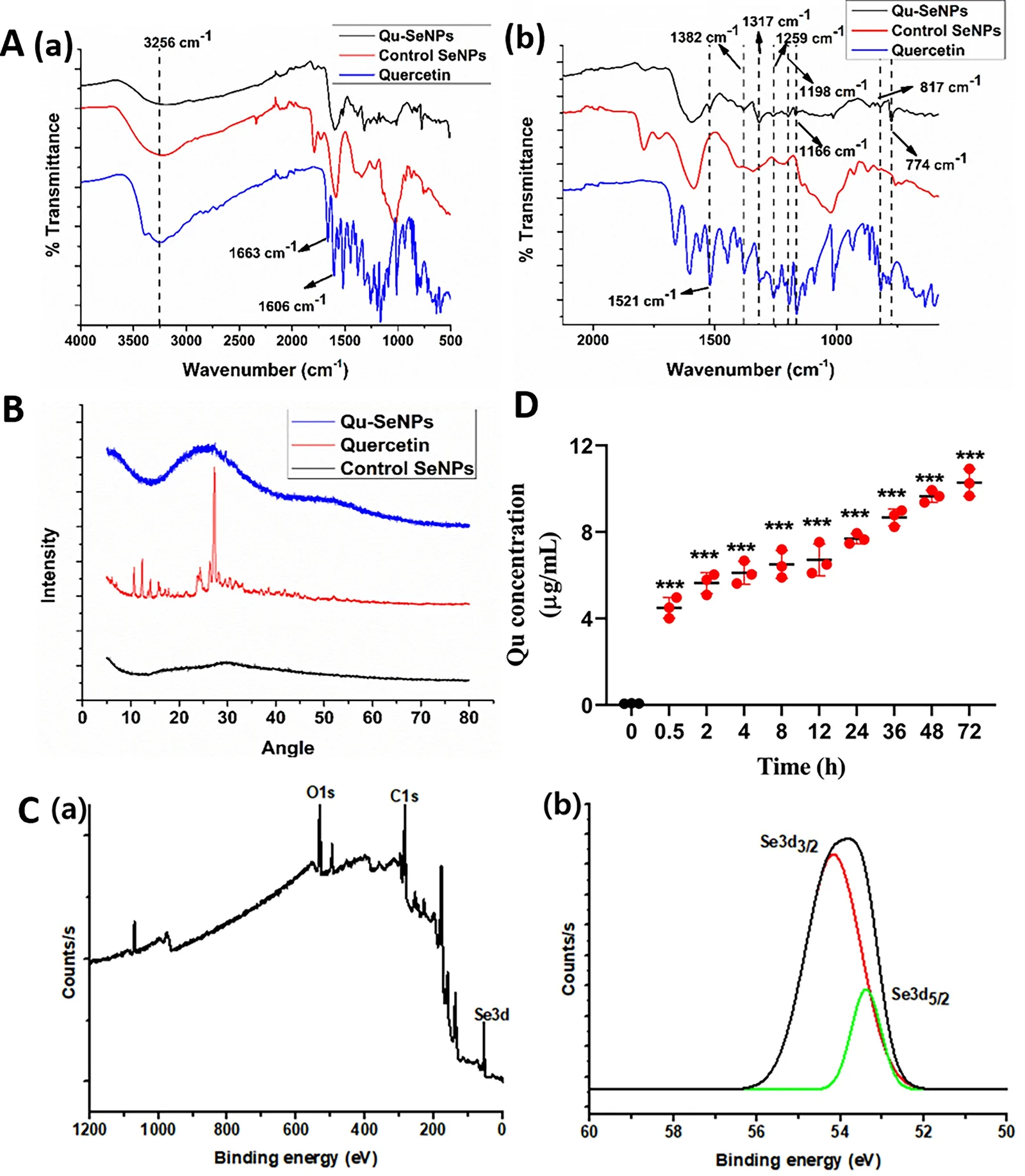
**Physicochemical characterization of Qu-SeNPs**. (**A**) Full scan (500 - 4000 cm^-1^) (a) and expanded region (500 - 2000 cm^-1^) FT-IR spectra and (B) XRD spectra of Qu, control SeNPs, and Qu-SeNPs. (**C**) Full XPS scan (a) and narrow XPS scan (b) of Qu-SeNPs. (**D**) Time-dependent release of Qu from Qu-SeNPs (n=3;. ***p<0.001 *vs.* 0 ng mL^-1^, one-way ANOVA with Dunnett’s multiple comparisons test).

The Qu-SeNPs were easily redispersed in deionized water and exhibited a hydrodynamic size of around 140.7 nm (Fig. 1B). In addition, the PDI of Qu-SeNPs was 0.32 (Fig. 1B), which indicates an acceptable narrow size range in nanoparticle compositions. In contrast, after centrifugation and redispersion, larger aggregates of control SeNPs were observed with 3500.6 nm hydrodynamic size, possibly due to Van Der Waals inter-particle attractions [30] (Supplementary Fig. S2), which may be attributed to the lack of any stabilizing agent. Further, compared to the neutral zeta potential of control SeNPs (i.e., 0.52 mV), the zeta potential of Qu-SeNPs was -20.63 mV (Fig. 1C), indicating a negative charge due to the presence of Qu. The TEM images also indicated that the dimensions of the flower-shaped Qu-SeNPs were smaller than 50 nm (Fig. 1D). Additionally, the selected area electron diffraction (SAED) patterns indicated that the Qu-SeNPs possess an amorphous phase (Fig. 1D). The scanning electron microscopy (SEM) pictures revealed comparable morphology and size of Qu-SeNPs to TEM images, accompanied by a rough surface, suggesting the existence of Qu on the surface of the nanoparticles (Fig. 1E). Additionally, the energy-dispersive X-ray spectroscopy (EDX) confirmed the presence of selenium in the Qu-SeNPs (Fig. 1E). In contrast to Qu-SeNPs, the aggregation in the control SeNPs was confirmed by the TEM images, while the SEM images of control SeNPs exhibited larger bare nanoparticles with smooth surfaces (Supplementary Fig. 2).

To identify the presence of Qu in the Qu-SeNPs, the FTIR spectra of Qu and Qu-SeNPs were recorded and compared (Fig. 2A). In the FTIR spectrum of Qu, the OH groups stretching was detectable at 3256 cm^-1^, whereas the OH bending of the phenol function was detectable at 1382 cm^-1^. In addition, the C=O aryl ketonic stretch absorption was evident at 1663 cm^-1^, along with C=C aromatic ring stretch bands at 1606 and 1521 cm^-1^. The in-plane bending band of C-H in aromatic hydrocarbon was also detectable at 1317 cm^-1^, and out-of-plane bending bands were evident at 817 and 774 cm^-1^. Further, peaks at 1259, 1198, and 1166 cm^-1^ were attributable to the C-O stretching in the aryl ether ring, the C-O stretching in phenol, and the C-CO-C stretching and bending in ketone, respectively. The characteristic peaks of Qu were also present in the spectrum of Qu-SeNPs but absent in the FTIR spectrum of control SeNPs.

The sodium selenite shows a crystalline XRD pattern with well-defined crystal structures and regular atom patterns (Supplementary Fig. S3A). Similarly, the XRD pattern of pristine Qu also shows its crystalline nature (Fig. 2B). However, both the Qu-SeNPs and control SeNPs showed an amorphous nature in the XRD pattern (Fig. 2B). XPS analysis has been employed to study the chemical composition and oxidation states of Qu-SeNPs. The selenium in the Qu-SeNPs was confirmed using XPS, as shown in Figure 2C. The peaks at 531 eV, 285 eV, and 54 eV were assigned to the O1s, C1s, and Se3d signals, respectively. In the narrow scan image, two peaks of Se3d, i.e., Se3d_3/2_ and Se3d_5/2_, signal peaks appeared at about 54.18 eV and 53.38 eV, respectively, with energy separation of about 0.8 eV. Similarly, control SeNPs also showed two peaks of Se3d, i.e., Se3d3/2 and Se3d5/2, signal peaks appeared at about 54.66 eV and 53.82 eV, respectively, with energy separation of about 0.84 eV (Supplementary Fig. S3B). These results are consistent with previous reports [28, 31, 32]. Further, the time-dependent release of Qu from Qu-SeNPs was assessed, which indicated that the significant release of Qu from Qu-SeNPs within 30 min of incubation and continued at least till 72 h of incubation, indicating weak non-covalent interaction, such as coordination bond [33] between Qu and SeNPs (Fig. 2D).

### Effect of Qu-SeNPs on ALP activity and other osteogenic markers of osteoblasts

**Figure 3.**
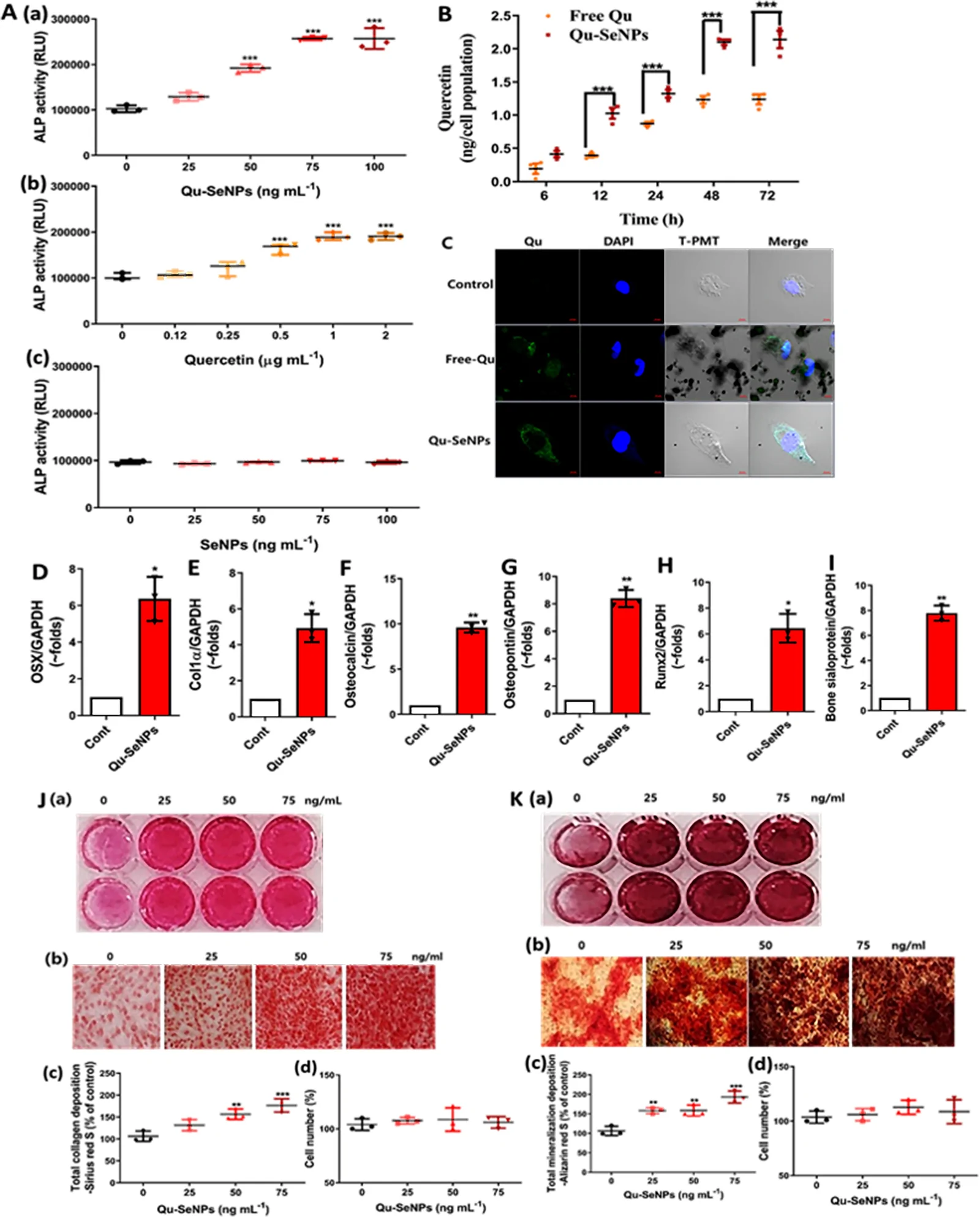
**Enhanced Qu-SeNPs uptake induces osteogenic activity in osteoblasts**. (**A**) (a-c) ALP activity of MC3T3-E1 cells treated with Qu-Se NPs, Qu and Se NPs, at various concentrations for 48 h (***p<0.001 *vs.* 0 ng mL^-1^, n=3; one-way ANOVA with Dunnett’s multiple comparisons test). (**B**) Time-dependent cellular uptake of free Qu and Qu-SeNPs. (n=3; ***p<0.001; 2way ANOVA with Sidak’s multiple comparisons) (C) Confocal images of cellular uptake of free Qu (1 µg mL^-1^) or Qu-SeNPs at equivalent Qu concentration (75 ng mL^-1^) after 12 h of incubation. (**D-I**) Real-time PCR analysis for the mRNA expression of osteogenic genes after treating Qu-SeNPs (75 ng mL^-1^) to MC3T3-E1 cells for 48 h. The results show a fold increase compared to GAPDH expression. The relative expression level is compared to the control (n=3; *p<0.05, **p<0.01; Paired t-test). MC3T3-E1 cells were administered Qu-Se NPs at the concentration shown in the figure, and after 7 days of treatment, collagen synthesis was evaluated by (J(a) & (b)) Sirius Red S staining and mineralization by (K(a) & (b)) Alizarin red staining. The extraction of stained cells was used to ascertain the quantification of collagen synthesis (J(c) & (d)) and mineralization (K(c) & (d)) (n=3; **p<0.01, ***p<0.001 *vs.* 0 ng mL^-1^; one-way ANOVA with Dunnett’s multiple comparisons test).

MC3T3 cells were treated with sub-cytotoxic concentrations of Qu-SeNPs, control SeNPs, and free Qu (supplementary Fig. S4), and ALP activity, one of the most reliable markers for osteogenic differentiation produced by osteogenic cells, was analyzed. It was observed that the Qu-SeNPs significantly increased the ALP activity at concentrations lower than free Qu (Fig. 3A). Moreover, it was observed that the control SeNPs did not affect the ALP activity at concentrations equivalent to Qu-SeNPs, indicating a lack of osteogenic property in SeNPs at selected concentration. Thus, SeNPs were removed from further biological activity experiments.

Further, since there is a correlation between the efficient accumulation of Qu and its osteogenic activity, to validate the enhanced cellular uptake of Qu in the cells treated with Qu-SeNPs, compared to cells treated with free Qu, the MC3T3 cells were time-dependently treated with free Qu or Qu-SeNPs (loaded with an equal amount of Qu) for 6 h, 12 h, 24 h, 48 h, and 72 h. The results demonstrated a time-dependent increment in the Qu uptake by the cells treated with both free Qu and Qu-SeNPs, where the maximum Qu uptake plateau was reached after 48 h (Fig. 3B). Additionally, confocal microscopic analysis was performed to determine the cellular distribution of Qu in the cells treated with free Qu and Qu-SeNPs (6 h, 12 h, 24 h, 48 h, and 72 h). As shown in Figure 3C, the free Qu-treated cells show aggregates of Qu (mainly on the outside of the cells). In contrast, the confocal images of the cells treated with Qu-SeNPs have a more evenly distributed green fluorescence signal of Qu compared to free Qu-treated cells, which suggests that the SeNPs act as carriers and improve the cellular uptake of Qu [34]. Further, the confocal images showing higher fluorescence signals in the cells corroborated with the quantification results of Qu content in the cells. These results strongly suggested the preferable attachment of Qu with SeNPs for enhanced uptake by bone cells.

The osteogenic effect of Qu-SeNPs was validated by assessing the mRNA expression levels of osteogenic markers, including the master regulator of osteogenesis (Osterix), early osteoblast differentiation markers (Col1α and Runx2), and terminal differentiation markers (Bone Sialoprotein, Osteopontin, and Osteocalcin) following a 48 h treatment of MC3T3-E1 cells with Qu-SeNPs. Since 75 ng mL^-1^ of Qu-SeNPs showed maximum induction of ALP activity of osteoprogenitors thus, this concentration was selected to study further the effect of Qu-SeNPs on osteoprogenitors. Relative to control, Qu-SeNPs stimulated the expression of osterix (~7-fold), col1a (~5-fold), Runx2 (~6-fold), OCN (~8-fold), OPN (~8-fold), and BSP (~8-fold) (Fig. 3D-I). Additionally, the effect of varying concentrations of Qu-SeNPs (0, 25, 50, and 75 ng mL^-1^) on terminal phases of differentiation, such as collagen synthesis and mineralization, was compared and analyzed. MC3T3-E1 cells treated with Qu-SeNPs (75 ng mL^-1^) for 7 days showed concentration-dependent induction of collagen synthesis and mineralization, as depicted by the Alizarin Red and alizarin red S staining (Fig. 3J&K).

### Qu-SeNPs activate WNT and BMP signaling pathways in osteoprogenitors

**Figure 4.**
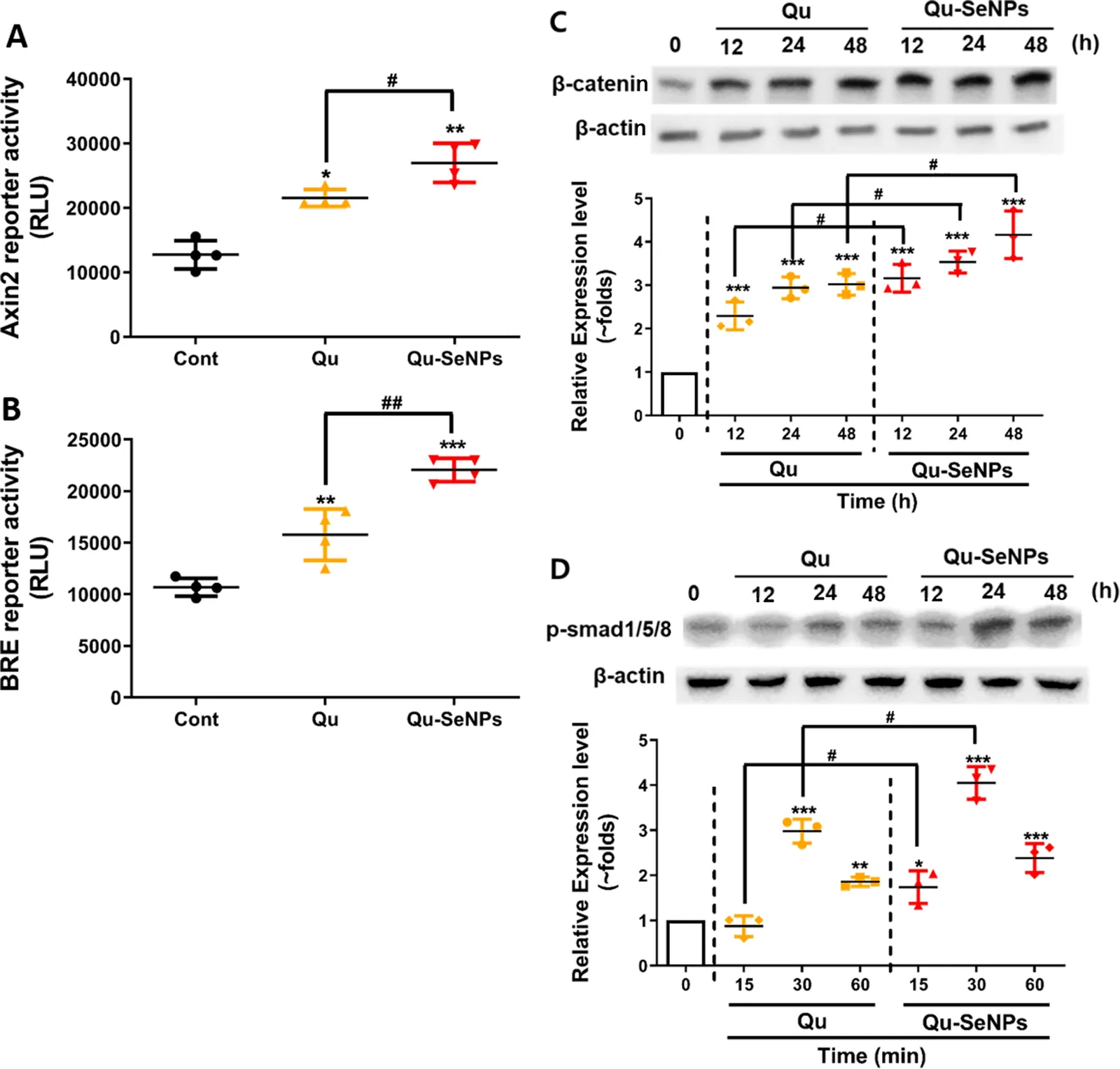
**Qu-SeNPs activate WNT and BMP signaling pathways in osteoblasts**. (**A**) Transiently transfected MC3T3-E1 cells with Axin-2 and (B) BRE reporter construct were treated free Qu (1 µg mL^-1^) or Qu-SeNPs at equivalent Qu concentration (75 ng mL^-1^) (n = 4; *p<0.05, **p<0.01, ***p<0.001 *vs.* control; one-way ANOVA with Dunnett’s multiple comparisons test, and ^#^p<0.05, ^##^p<0.01; unpaired t-test). Luciferase activities were assessed in cell lysates after 48 h and normalized to Renilla luciferase activity, as detailed in the Materials and Methods section. MC3T3-E1 cells were treated with free Qu (1 µg mL^-1^) or Qu-SeNPs at an equivalent Qu concentration (75 ng mL^-1^) for the time indicated in the figure. Protein lysates were subsequently collected. Western blotting revealed the stabilization of (C) β-catenin and the (D) phosphorylation of Smad 1/5/8 molecules. β-actin was a loading control and normalized for densitometric analysis of Western blot bands. The relative expression level is compared to the control (n = 3; *p<0.05, **p<0.01, ***p<0.001 *vs.* 0 h; one-way ANOVA with Dunnett’s multiple comparisons test, and ^#^p<0.05 *vs.* free Qu at similar time points; unpaired t-test.

To observe the effect of Qu-seNPs in osteoblast differentiation, the activation of WNT and BMP signaling pathways by Qu-SeNPs in osteoblasts was examined. Axin-2 or BRE luciferase reporter construct were transfected for 24 h to MC3T3-E1 cells and treated with Qu (1 µg mL^-1^) or Qu-SeNPs (75 ng mL^-1^). After 48 h of treatment, the effect was analyzed. Axin-2 and BRE luciferase activity was increased both by Qu (2.5 and 1.5 folds) and Qu-SeNPs (3 and 2.5 folds) compared to control, respectively. However, a Qu-SeNPs concentration of 75 ng mL^-1^ resulted in a more significant increase in reporter plasmid activity than 1 µg mL^-1^ (Fig. 4A&B). MC3T3-E1 cells were treated at a concentration of 1 µg mL^-1^ of Qu and 75 ng mL^-1^ of Qu-SeNPs for 12, 24, and 48 h. Both Qu and Qu-SeNPs enhanced β-catenin stabilization from 12 to 48 h. Nonetheless, a notable enhancement in β-catenin stabilization was seen using Qu-SeNPs in comparison to Qu (Fig. 4C). Also, treatment of 1 µg mL^-1^ of Qu and 75 ng mL^-1^ of Qu-SeNPs for 15, 30, and 60 min revealed that smad1/5/8 molecules were maximum phosphorylated at 30 min of exposure (Fig. 4D).

### Qu-SeNPs inhibit osteoclastogenesis

**Figure 5.**
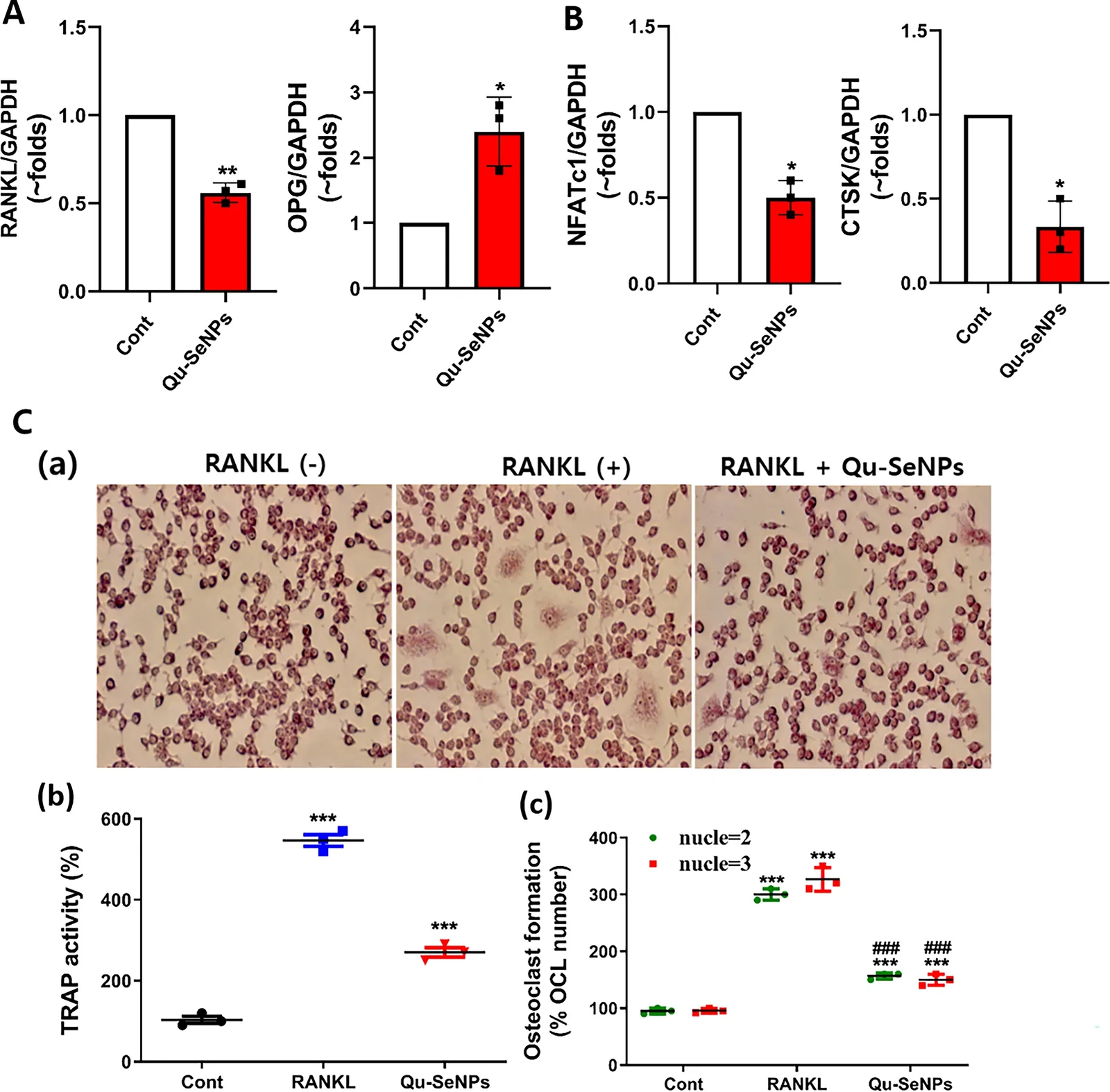
**Qu-SeNPs inhibit osteoclastogenesis and osteoclast formation**. (**A**) Real-time PCR analysis for the mRNA expression of RANKL, OPG after treating Qu-SeNPs (75 ng mL^-1^) to MC3T3-E1 cells for 48 h. (**B**) Real-time PCR analysis for the mRNA expression of NFATc1 and CTSK after treating Qu-SeNPs (75 ng mL^-1^) to RAW 264.7 cells (stimulated with RANKL (50ng mL^-1^)) for 48 h. The results show a fold increase compared to GAPDH expression. The relative expression level is compared to the control (n=3; *p<0.05, **p<0.01, ***p<0.001 *vs*. control; Paired t-test). (**C**) RAW 264.7 cells were treated with RANKL (50 ng mL^-1^) for 2 days, followed by treatment with Qu-Se NPs (75 ng mL^-1^). After 7 days, Trap staining was performed to evaluate the trap-positive cells (40X). TRAP activity % and % osteoclast (OCL) number is compared to control (n=3; 2way ANOVA with Tukey’s multiple comparisons test) Three independent experiments yielded similar results (n=3; ***p < 0.001 *vs.* respective control, and ^###^p < 0.001 *vs.* respective RANKL-treated groups; 2way ANOVA with Tukey’s multiple comparisons test).

To evaluate the ability of Qu-SeNPs to inhibit osteoclastogenesis, Qu-SeNPs were treated to MC3T3-E1 cells for 48 h, and the mRNA expression of RANKL and OPG were evaluated. Qu-SeNPs inhibited the mRNA expression of RANKL while enhancing the OPG in osteoprogenitors (Fig. 5A). Treatment of Qu-SeNPs to differentiate (treated with RANKL (50 ng/ml) for 3 days) murine macrophage cell line RAW 264.7 cells for 48 h reduced the mRNA expression of NFATc1 and cathepsin K (CtsK) (Fig. 5B). The effect of Qu-SeNPs on inducing osteoclastogenesis was further examined by evaluating the TRAP-positive cells. Following a 3-day stimulation with RANKL, Qu-SeNPs (75 ng mL^-1^) were administered to RAW 264.7 cells for 7 days, and TRAP-positive cells were identified using TRAP staining as outlined in the materials and methods. RANKL-treated samples exhibited enhanced osteoclastogenesis (increased TRAP-positive cells) via the induction of multinucleated cell forms (osteoclasts) relative to the control group. However, low osteoclast formation was observed in Qu-SeNPs treated RAW 264.7 cells (Fig. 5C).

### Qu-SeNPs involve miR-206/Cx43 pathway to mediate osteogenic activity

**Figure 6.**
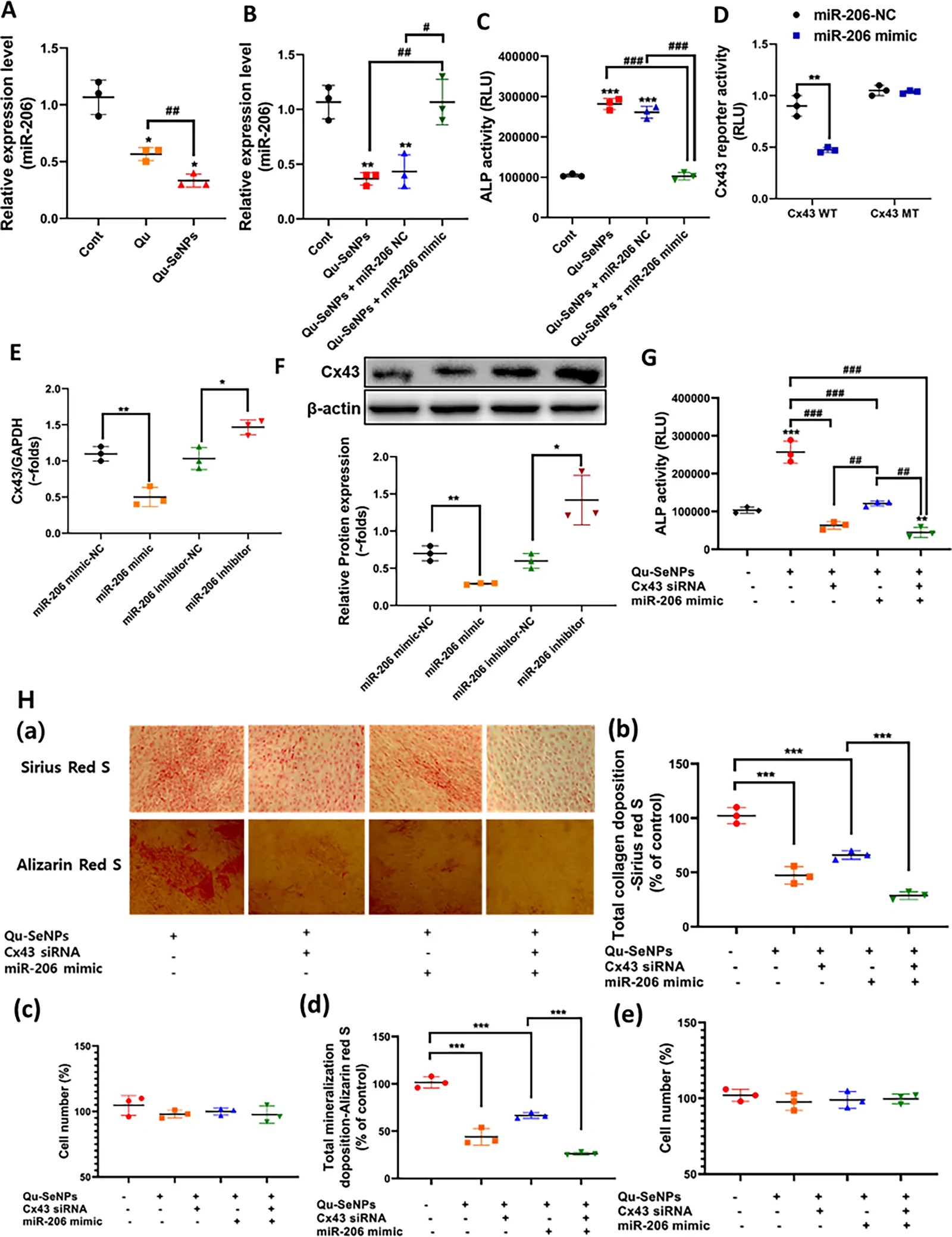
**Qu-SeNPs stimulate osteogenesis via miR-206/connexin 43 (Cx43) pathway**. (**A**) Real-time PCR analysis for the miR-206 expression after treating Qu (1 µg mL^-1^) or Qu-SeNPs (75 ng mL^-1^) to MC3T3-E1 cells for 72 h. The results show a fold increase compared to U6 expression (n=3; *p<0.05, *vs*. control; Paired t-test, and ^##^p < 0.01; unpaired t-test). (**B**) Real-time PCR analysis for the miR-206 expression in MC3T3 E-1 cells treated with Qu-SeNPs (75 ng mL^-1^) along with miR-206 mimic or miR-206 NC. Relative expression level is compared to control (n=3; **p<0.01, *vs*. control; one-way ANOVA with Dunnett’s multiple comparisons test, and ^#^p < 0.05, ^##^p < 0.01; unpaired t-test). (**C**) ALP activity of MC3T3 E-1 cells treated with Qu-SeNPs (75 ng mL^-1^) along with miR-206 mimic or miR-206 NC (n=3; ***p<0.001 *vs.* control; one-way ANOVA with Dunnett’s multiple comparisons test, and ^###^p < 0.001; unpaired t-test). (**D**) Luciferase reporter activity of MC3T3 E-1 cells transfected with wild type Cx43 sequence (Cx43 WT) pGL3 plasmid or mutant Cx43 sequence (Cx43 MT) pGL3 plasmid) and treated with miR-206 mimic (n=3; **p<0.01; 2way ANOVA with Sidak’s multiple comparison). (E & F) Relative mRNA and protein expression of endogenous Cx43 in MC3T3E-1 cells treated with miR-206 mimic or miR-206 inhibitor. miR-206 NC or miR-206 inhibitor-NC were used as control (n=3; *p<0.05, **p<0.01; unpaired t-test). (**G**) ALP activity of MC3T3 E-1 cells transfected with Cx43 siRNA, Qu-SeNPs treated alone or along with miR-206 mimic (n=3; ***p<0.001 *vs.* control; and ^##^p < 0.01, ^###^p < 0.001; one-way ANOVA with Tukey’s multiple comparisons test). (**H**) Representative pictures of Sirius Red S and Alizarin red S staining of MC3T3 E-1 cells treated with Cx43 siRNA and miR-206 mimic in osteogenic medium having Qu-SeNPs for 7 days (n=3; ***p<0.001; one-way ANOVA with Tukey’s multiple comparisons test).

Treatment of Qu or Qu-SeNPs to MC3T3 E-1 cells for 48 h showed a downregulation of the mRNA expression of miR-206 in the osteoprogenitor cell line (Fig. 6A). Overexpression of miR-206 in MC3T3 E-1 cells with miR-206 mimic, treated with Qu-SeNPs, restored the mRNA expression levels of miR-206 in osteoblasts (Fig. 6B). However, ALP activity induced by Qu-SeNPs was inhibited by the elevated expression of miR-206 in osteoblasts, confirming the negative effect of miR-206 on the osteogenic marker like ALP activity (Fig. 6B & C). MC3T3 E-1 cells were treated with miR-206 mimic-negative Control (miR-206 NC) as a control.

The wildtype reporter gene activity of Cx43 was significantly inhibited by exogenous treatment of miR-206. However, when there was a mutation in the seed site of Cx43, the repression by exogenous miR-206 was abolished entirely (Fig. 6D). This demonstrates that miR-206 directly regulates Cx43 gene expression by binding to the mutated seed site within its 3′UTR. To further investigate the potential regulatory role of miR-206 on osteogenic differentiation through its interaction with the Cx43 gene, we assessed the expression of the Cx43 gene by RT-PCR in MC3T3 E-1 cells following transfection with either miR-206 mimics or a miR-206 inhibitor. The results indicated that the mRNA expression level of the Cx43 gene was significantly reduced in osteoblasts overexpressing miR-206 compared to the miR-206 mimic-NC group. Conversely, the expression level of the Cx43 gene was significantly increased in the miR-206 inhibitor group (Fig. 6E). Similarly, Cx43 protein expression corresponded to the mRNA results, proposing that miR-206 regulates osteogenic differentiation by selectively targeting the Cx43 gene in MC3T3 E-1 cells (Fig. 6F).

To examine whether Qu-SeNPs involve miR-206/Cx43 pathway in promoting osteogenic differentiation, Cx43 siRNA was transfected to MC3T3 E-1 cells alone or in combination with miR-206 mimic and treated with Qu-SeNPs for 48 h for ALP activity and 7 days for evaluating any effect on collagen synthesis and mineralization. As shown in Figure 6 G&H, silencing Cx43 reduced the miR-206 mimic-mediated and Qu-SeNPs-mediated increase in the ALP activity, collagen synthesis, and mineralization.

### Hydrogel-embedded Qu-SeNPs improve bone regeneration in mice drill hole model

**Figure 7.**
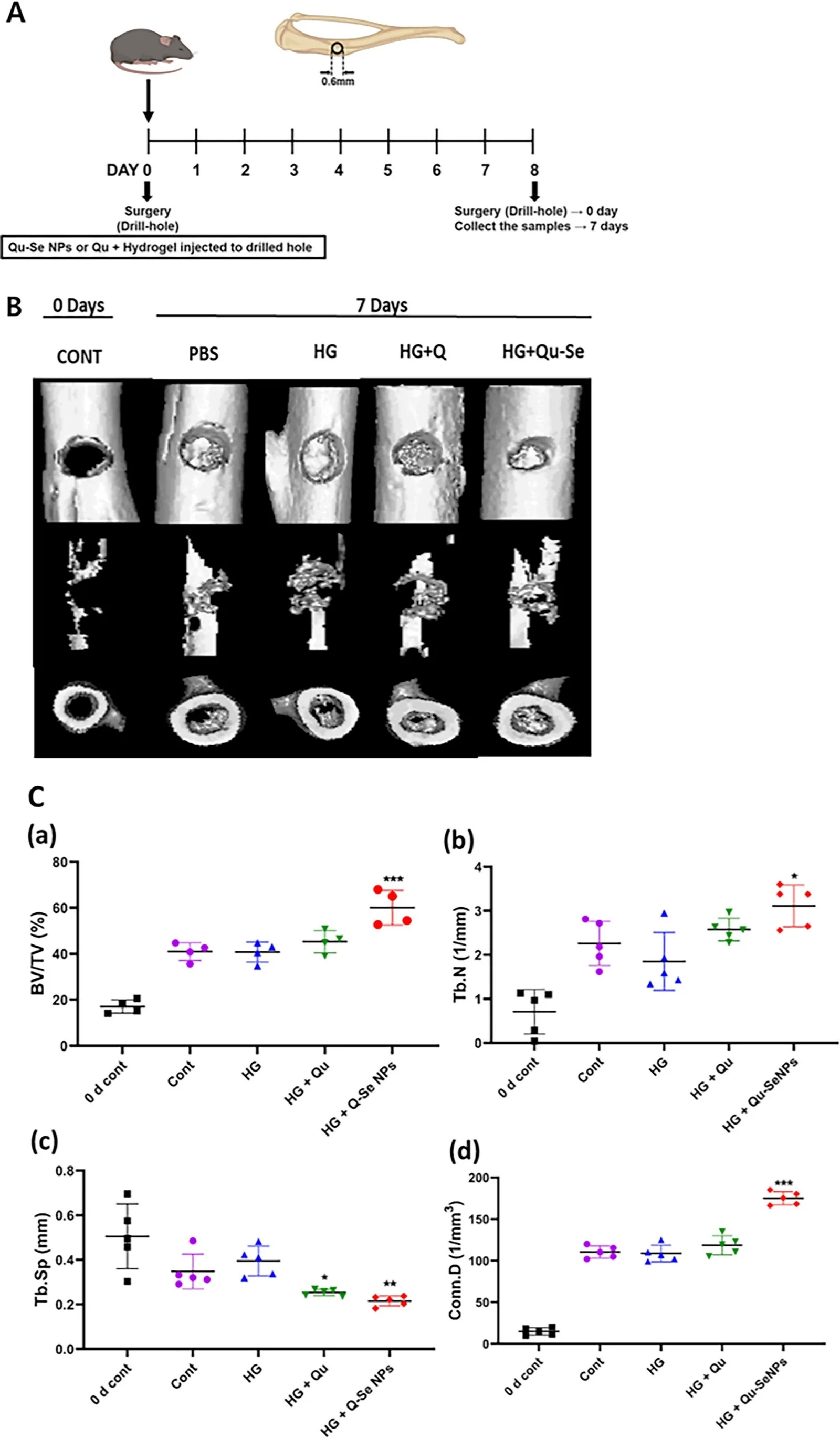
**Hydrogel-mediated delivery of Qu-SeNPs in mice drill hole model improves bone regeneration**. (**A**) Schematic experimental plan for the hydrogel-based delivery of Qu and Qu-Se NPs to drill holes in bone defect mice model. (**B**) Representative 3D images by microCT demonstrating bone regeneration in drill hole defect in mice tibiae after 7 days of Qu or Qu-SeNPs treatment as described in the material and method section. (**C**) Analysis of microCT showing comparative results for bone volume fraction (BV/TV), trabecular number (Tb.N), trabecular separation (Tb.Sp.), and connectivity density (Conn.D) (n=5; *p< 0.05, **p < 0.01, ***p < 0.001 *vs.* control; one-way ANOVA with Dunnett’s multiple comparisons test).

The surgical procedure was performed in mice to create a hole (0.6 mm in diameter) through the posterior and anterior cortices of the tibiae. Subsequently, mice were administered Qu or Qu- embedded in hydrogel for 7 days as described in the material and methods (Fig. 7A). Biocompatible hydrogels have great potential as solid scaffolding materials for bone regeneration. Hydrogels, composed of water (up to 99%) and a hydrophilic polymer, are ideal for cell survival in physiological environments with minimal invasive requirements [34] and are used in bioengineering applications like drug delivery and tissue regeneration [35, 36]. The injectable hydrogel can change from a sol to a gel on the spot in response to changes in temperature, pH, or the redox property of the environment [35]. Thus, hydrogel is intriguing for future therapeutic applications that avoid open surgery. In this study, commercially available hydrogel, VitroGel hydrogel Matrix (VitroGel® (TheWell Bioscience, NJ, USA) — a xeno-free functional hydrogel matrix) was utilized for the localized delivery of Qu-SeNPs to the site of bone defect at the tibiae drill hole mice model.

After 7 days of treatment of hydrogel-embedded Qu-SeNPs to the drill hole site in the tibiae of mice, the tibiae were harvested, and micro-CT analysis was performed. Micro-CT enables the real-time observation of changes in bone structure, provides a clear view of the healing process, and can detect even minor discrepancies between different groups [37]. Control (0 days) here denotes the creation of a drill hole model, and the tibiae was harvested on the same day to symbolize bone defect in the tibiae, while PBS represents the administration of the only PBS without any hydrogel or Qu or Qu-SeNPs for 7 days and reflects the typical course of healing for bone.

3D radiological images by micro-CT analysis revealed an increase in the mineralized callus at the defect and intra-medulla region of the Qu-SeNPs treated drill hole area compared to control (PBS) or only hydrogel or hydrogel Qu administered at the bone defect site (Fig. 7B). A normal controlled bone regeneration healing process was observed in the control group treated with only PBS for 7 days. Bone volume (BV) is a metric used to quantify the number of tetrahedrons inside the structure based on the triangulated surface. On the other hand, total volume (TV) refers to the volume of the whole sample being analyzed. BV/TV evaluates the relative augmentation of bone volume within the affected bone region. At the same time, the trabecular number (Tb. N) is calculated by taking the reciprocal of the average distance between the mid-axes of the studied structure. Tb. N quantifies the degree of space occupancy inside the evaluated structure. The BV/TV changes and Tb. N in the drill-hole locations exhibited a substantial increase in the intramedullary region of hydrogel Qu-SeNPs treated groups compared to the PBS-treated group. While a small, non-significant increment was observed in the hydrogel-Qu-treated group (Fig. 7C (a & b)). The density with which the matrix occupies the space is defined by trabecular space (Tb.Sp.). Administration of Qu-SeNPs-loaded in hydrogel significantly decreased the Tb.Sp. in the drill hole bone defect site in the tibae compared to the PBS-treated group (Fig. 7C (c)). However, a significant decrease in Tb.Sp was observed in the hydrogel Qu-treated groups. The connectivity density (Conn.D) is a quantitative measure of the number of connections existing in the analyzed structure. It indicates the level of density at which the structure has been generated. Compared to the PBS group, the group treated with hydrogel Qu-SeNPs exhibited a substantial increase in the density of connections generated at the bone defect site. This increase was twice as much as the PBS group, as shown in Figure 7C (d).

## DISCUSSION

Qu's cellular uptake is limited due to its hydrophobic properties. However, the cellular uptake of Qu can be increased using nanoparticles, either by loading Qu inside the nanoparticles or by conjugating Qu with metal/non-metal nanoparticles. This study used Qu to synthesize/functionalize SeNPs, facilitating Qu's cellular uptake and resulting in Qu's enhanced biological activities. The instability of non-functionalized SeNPs has been previously reported, which renders SeNPs prone to aggregation and transforms them into inert forms in an aqueous medium [38, 39]. Surface functionalization has been an effective measure to promote the dispersity of SeNPs, improving their stability in aqueous solution [39]. Here, the formation and stabilization of SeNPs in the presence of Qu is believed to stem from the nucleation of Se in the solution, followed by particle growth, which is later stabilized by Qu coating, providing steric hindrance and preventing the aggregation of Qu-SeNPs [28, 40]. Therefore, the stability of SeNPs confirmed the presence of Qu as a stabilizing and functionalizing agent for SeNPs. The synthesis of Qu-SeNPs was further confirmed by the small size of Qu-SeNPs, which contributes to its amorphous nature (Fig. 1 & Fig. 2), possibly because at nano dimensions, the sodium selenite may lose the long-range order in bulk material, resulting in the disordered arrangement of atoms. Also, when associated with SeNPs, the Qu can lose its crystalline form due to its adsorption onto the SeNPs.

It has been known that when stabilized by a bioactive agent, the inorganic nanoparticles can facilitate the cellular uptake of the stabilizing agent [41]. Expectedly, a significantly higher accumulation of Qu was observed in the cells treated with Qu-SeNPs compared to free Qu-treated cells (Fig. 3), confirming that SeNPs facilitate the cellular uptake of Qu. As observed by confocal imaging (Fig. 3), the aggregation of Qu at a few localized areas in the cells treated with free Qu suggests a less uniform intracellular presence of Qu, which might result in reduced therapeutic efficacy. This aggregation of free Qu could be due to its hydrophobic properties, which might have prevented its cellular uptake. Our results are in accordance with the previous report, which showed aggregates of another flavonoid, i.e., curcumin, outside the cells, while curcumin-loaded copolymer micelles showed a uniform signal of curcumin inside the cells [42]. As a result of a more uniform and consistent cellular distribution of Qu when carried out with SeNPs, improved therapeutic activity can be assumed. This explains the enhanced ALP activity and osteoinductive properties of Qu-SeNPs in treated osteoprogenitors (Fig. 3). The increased expression of the early and late differentiation markers suggests that Qu-SeNPs have the potential to stimulate bone formation and regeneration.

WNT and BMP signaling pathways are crucial in regulating osteoblast differentiation and bone formation [43]. The activation of the WNT signaling pathway stabilizes β-catenin in the nucleus, where it subsequently interacts with the TCF/LEF family of DNA-binding proteins, therefore regulating WNT-mediated target genes involved in osteogenesis [43]. Moreover, the stimulation of the BMP signaling pathway results in the downstream phosphorylation of smad1/5/8 molecules, which subsequently facilitates the transcriptional control of osteogenic genes [44]. Therefore, following the treatment of Qu and Qu-SeNPs to MC3T3-E1 cells, the western blotting technique was utilized to detect the stabilization of β-catenin molecules and the phosphorylation of smad1/5/8 molecules. Compared to Qu, Qu-SeNPs elicited more phosphorylation of smad1/5/8 molecules and enhanced stability of β-catenin (Fig. 4). Taking together, Qu-SeNPs elicit osteogenic activity in osteoblasts via activating WNT and BMP signaling pathways and demonstrate higher stimulatory ability than Qu.

Osteoprotegerin (OPG) and the receptor activator of nuclear factor κB ligand (RANKL) form a complicated controlling system during bone resorption. The ratio of RANKL to OPG is the primary regulatory factor for bone resorption [45]. Osteoblasts are known to secrete RANKL and its decoy receptor, OPG, to contribute to osteoclastogenesis. A decreased RANKL to OPG ratio shows the ability of Qu-SeNPs to inhibit osteoclastogenesis and favor osteoblastogenesis (Fig. 5).

Thus, inhibiting RANKL secretion from the osteoblasts helps regulate bone resorption and restore the bone remodeling process usually found disturbed in bone diseases [46]. RANKL is considered a primary factor responsible for inducing osteoclast precursors' differentiation into osteoclasts [2]. It stimulates osteoclast differentiation by activating its receptor, RANK, and modulating gene expression through NFATc1. NFATc1 controls the activation of many genes and is required for osteoclast differentiation, such as TRAP, c-Fos, and CTSK [47]. Thus, the reduced expression of NFATc1 and CTSK after Qu-SeNPs treatment demonstrated the inhibitory effect of Qu-SeNPs on the RANKL-stimulated osteoclast differentiation process (Fig. 5). In addition, reduction in TRAP-positive cells also showed the inhibitory effect of Qu-SeNPs on RANKL-induced osteoclastogenesis (Fig. 5). An increased level of RANKL is related to bone diseases like osteoporosis as it promotes osteoclasto-genesis and causes rapid osteoclast-mediated bone to break down or bone loss. High levels of RANKL suppress or delay the anabolic effect of drugs stimulating bone formation and, thus, bone healing or regeneration. Hence, an agent that prevents or inhibits RANKL-induced osteoclastogenesis can promote bone healing or regeneration while restoring bone formation in bone diseases like osteoporosis.

Micro RNAs (miRNAs), a new class of short noncoding RNAs, regulate gene expression by degrading mRNA or impeding translation. Studies in recent years have linked miRNAs to various biological processes, such as cell proliferation, differentiation, activity, apoptosis, and metabolism [48]. Recently, it has been identified that Qu induces osteogenic differentiation in bone marrow stromal cells by regulating the miR-206/Cx43 pathway [49]. Previously identified as a muscle-specific miRNA, miR-206 is downregulated during osteoblast differentiation, and overexpression reduces the differentiation process of osteoblasts by targeting Cx43 *in vitro* and *in vivo* [50], implicating negative regulation of miR-206 on osteogenic differentiation.

Thus, whether the synthesized Qu-SeNPs also involve similar miRNA regulation to Qu, expression levels miR-206 in MC3T3 E-1 cells were analyzed. Since the ALP activity of osteoblasts was upregulated by Qu-SeNPs and expression levels of miR-206 were found downregulated, it was presumed that the miR-206 might be involved in Qu-SeNPs enhanced osteogenic stimulation in osteoblasts (Fig. 6). Previous studies have observed Cx43 as the target gene for the miR-206 in C2C12 mesenchymal progenitor cells [50, 51], and rat bone marrow stromal cells [49, 52]. To confirm Cx43 as the target gene for miR-206 in MC3T3 E-1 osteoprogenitors cells, luciferase reporter constructs under transcriptional control of the wildtype CX43 3′ UTR or mutant CX43 3′UTR were transfected with miR-206 mimic or control (non-targeting, miR-NC). Results showed that silencing Cx43 prevented the expected increase in ALP activity, collagen synthesis, and mineralization, as evidenced in miR-206 mimic and Qu-SeNPs treated MC3T3 E-1 cells (Fig. 6). These findings indicate that higher levels of miR-206 expression are associated with the inhibitory effect of Qu-SeNPs-induced osteogenesis, which depends on the accumulation of Cx43 expression in osteoblasts. Recently, in rat BMSCs, the expression of Cx43 has been shown to modulate bone mass and bone mineral density by activating the WNT signaling pathway [52]. Thus, it appears that synthesized Qu-SeNps involves miR-206/Cx43 pathway to activate the WNT signaling pathway as observed by the increased stability of β-catenin after Qu-SeNps treatment in MC3T3 E-1 cells (Fig. 4A & C) and is able to induce osteogenic activity in osteoprogenitors. Moreover, during the osteoblast differentiation process, a cross-talk between WNT and BMP signaling pathways plays a crucial role, and WNT signaling is upstream of BMP signaling in osteoblasts [53]; hence, it might be possible that the miR-206/Cx43 pathway is responsible for the Qu-SeNPs mediated WNT signaling activation which then activates BMP signaling pathway in osteoblasts to induce osteogenic activity in osteoblasts.

The drill-hole bone defect model in mice was used to investigate the bone regeneration ability of Qu-SeNPs *in vivo*. Several research studies have used the drill-hole bone defect model to evaluate the bone-forming mechanisms implicated in bone repair, such as during fracture healing or delayed bone formation in osteoporosis [24, 54, 55]. These models are primarily used for testing the systemic administration of drugs or evaluating the impact of a particular gene alteration on bone regeneration, owing to the limited dimensions of the hole. By the third day following surgery, the drill site in the bone defect mice model usually consists of a proliferating and differentiating population of osteoblasts, osteoclasts, and chondrocytes that usually are from migrated mesenchymal cells and contribute to new bone formation [24].

*In vivo*, bone regeneration analysis supports the results obtained *in vitro* and highlights the enhanced bone regeneration ability of Qu-SeNPs over Qu (Fig. 7). This could be possible because of the rapid release of Qu from Qu-SeNPs owing to weak bonds between Qu and SeNPs, which might ensure that the maximum concentration of Qu is available immediately after application, resulting in an enhanced osteogenic effect. The micro-CT data analysis revealed that Qu treatment improved bone regeneration parameters, but the results were insignificant compared to those of Qu-SeNPs against control. Taken together, *in vitro* and *in vivo* results show the enhanced bone regeneration ability of synthesized Qu-SeNPs over free Qu.

## Conclusion

In conclusion, our research offers significant data endorsing the therapeutic effectiveness of the synthesized bioactive Qu-SeNPs composite in promoting bone formation and regeneration. The meticulously synthesized Qu-SeNPs composite showed promising stable characteristics and biological activities that enhance osteogenesis and inhibit osteoclastogenesis, making them a potential candidate for therapeutic applications in bone regeneration. Flavonoid-encapsulated biomaterials can significantly enhance bone defect healing; nonetheless, it is essential to augment the overall efficacy of these flavonoid-loaded bone repair biomaterials to enhance flavonoid bioavailability and expand options for bone defect remediation.

In our study, integrating Qu with SeNPs not only improved the stability and bioavailability of Qu but also leveraged its beneficial properties in promoting bone health. Our extensive *in vitro* and *in vivo* research demonstrate that cellular uptake of Qu-SeNPs compared to Qu was highly efficient and increased osteoprogenitors differentiation markers' expression, promoting bone formation. Moreover, compared to Qu mechanistically, Qu-SeNPs demonstrated heightened activation of bone formation-promoting signaling pathways like WNT and BMP signaling pathways in osteoprogenitors to induce osteogenesis. Qu-SeNPs also utilized the miR-206/Cx 43 pathway in MC3T3 E-1 cells to induce osteogenesis. miR-206/Cx 43 signaling axis has been shown to activate the WNT signaling pathway in rat BMSCs and thus suggests that Qu-SeNPs might also involve this axis to activate the WNT signaling pathway in MC3T3 E-cells. *In vivo*, studies in mice (drill hole bone defect mice model) showed increased bone defect healing properties of hydrogel-mediated delivery of Qu-SeNPs to the bone defect site in tibiae. The localized administration of Qu nanoformulations enhances therapeutic effectiveness at reduced dosages by enhancing Qu bioavailability, stability, and targeted delivery to the bone defect site. Using Qu-SeNPs as a rapid-releasing composite, which enhances the bioavailability of Qu for localized bone regeneration material, exhibits significant advantages for dynamic regeneration processes like fracture healing or coating bone grafts or implants where progressive bone regeneration is required. Moreover, localized delivery enables a continuous and regulated release of Qu, enhancing its therapeutic efficacy while mitigating possible adverse effects and decreasing the amount needed for effective therapy. However, further studies can clinically evaluate the cell population dynamics and signaling pathways regulated by Qu-SeNPs in clinical settings, which can help fully exploit the promise of Qu-SeNPs for a therapeutic approach to bone regeneration in aging-related bone diseases like osteoporosis and associated fractures. Additional research on patients with bone defects would be required to ascertain if outcomes seen in animal models can be replicated in people.

## Supplementary Materials

The Supplementary data can be found online at: www.aginganddisease.org/EN/10.14336/AD.2025.0025.
