# Supplementary material for: Bone Regeneration Enhanced by Quercetin-Capped Selenium Nanoparticles via miR206/Connexin43, WNT, and BMP signaling pathways
Source: Aging Dis. 2025 Feb 28;17(1):530–48. doi: 10.14336/AD.2025.0025 (PMC12727087; doi:10.14336/AD.2025.0025)
Supplement: Supplementary file 1 — The Supplementary data can be found online at: www.aginganddisease.org/EN/10.14336/AD.2025.0025. [file AD-17-1-530-s.pdf]

SUPPLEMENTARY DATA

Bone Regeneration Enhanced by Quercetin-Capped Selenium Nanoparticles via miR206/Connexin43, WNT, and BMP signaling pathways

Garima Sharma, Yeon Hee Lee, Jin-Chul Kim, Ashish Ranjan Sharma, Sang-Soo Lee

© 2025. Sharma G et al. Published online at http://www.aginganddisease.org/EN/10.14336/AD.2025.0025

SUPPLEMENTARY DATA

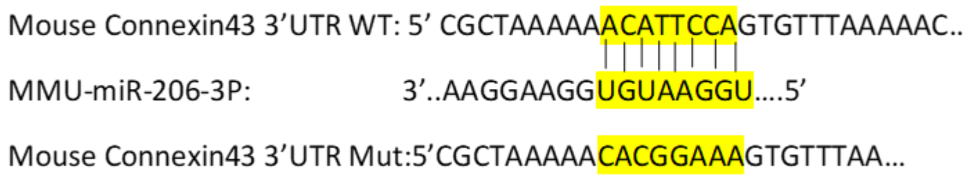

Supplementary Figure 1. Cx43: Sequences of wildtype (Cx43 WT) or mutant (Cx43 MT). miR-206 is complementary to the 3'UTR of Cx43 mRNA. Yellow sequences highlighted represent the altered nucleotides in the mutant reporter plasmid compared to wild type Cx43 sequence.

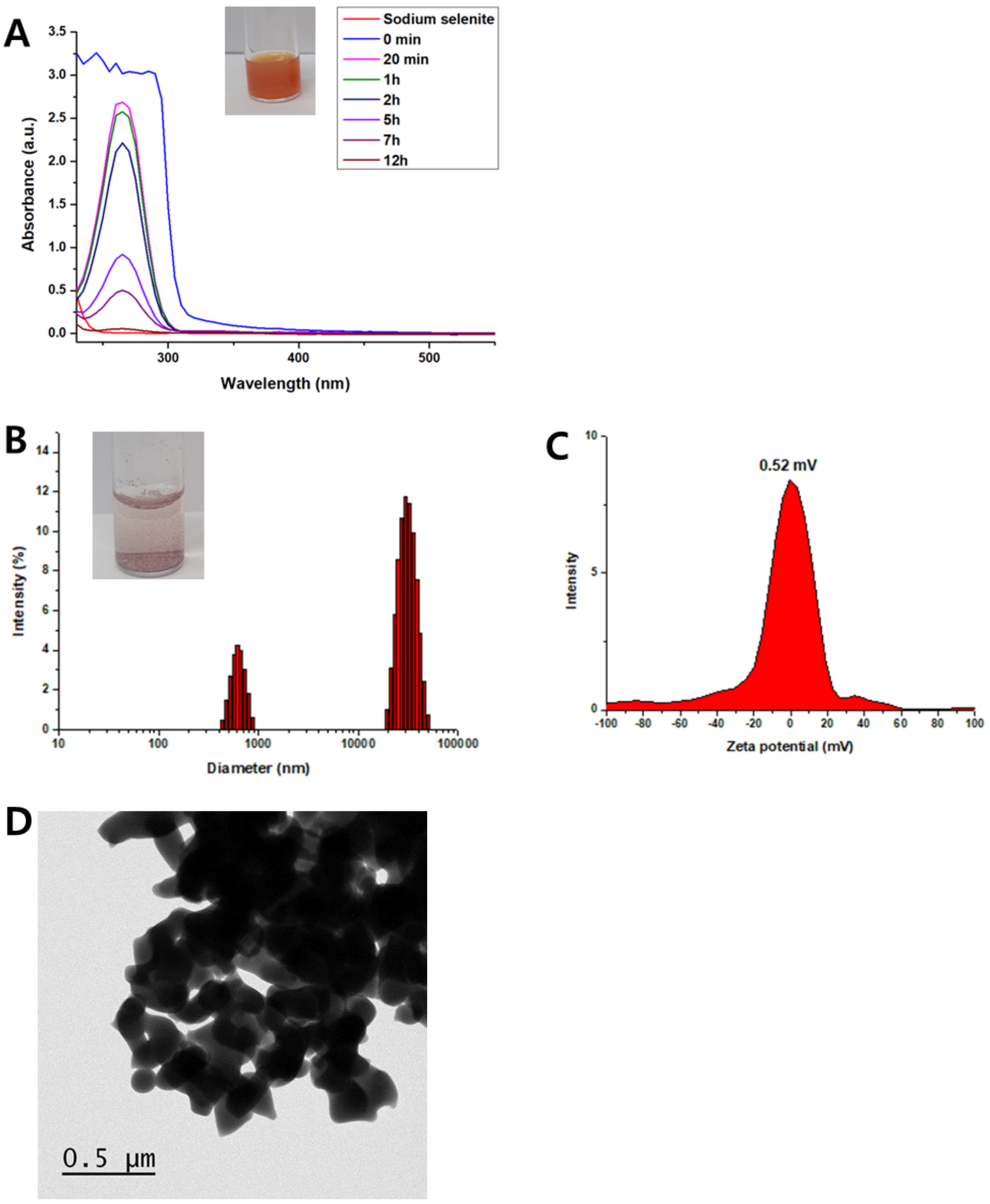

© 2025. Sharma G et al. Published online at http://www.aginganddisease.org/EN/10.14336/AD.2025.0025

SUPPLEMENTARY DATA

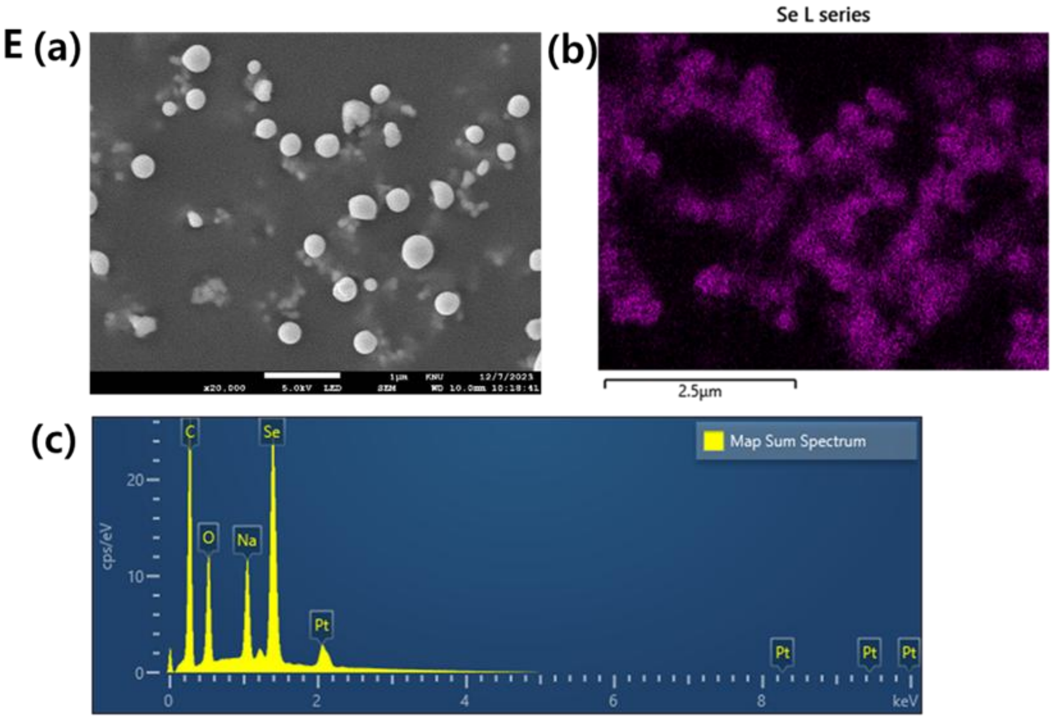

Supplementary Figure 2. Size and morphological characterization of SeNPs. (A) UV-vis absorption spectra of sodium selenite, and time-dependent synthesis of SeNPs (inset showing the digital image of SeNPs after synthesis). (B) Hydrodynamic size (inset showing the digital image of centrifuged and re-disperesed SeNPs), (C) Zeta potential, (D) TEM (a) and SAED (b) images, (E) SEM image (a) and EDX analysis (b & c) of Qu-SeNPs.

© 2025. Sharma G et al. Published online at http://www.aginganddisease.org/EN/10.14336/AD.2025.0025

SUPPLEMENTARY DATA

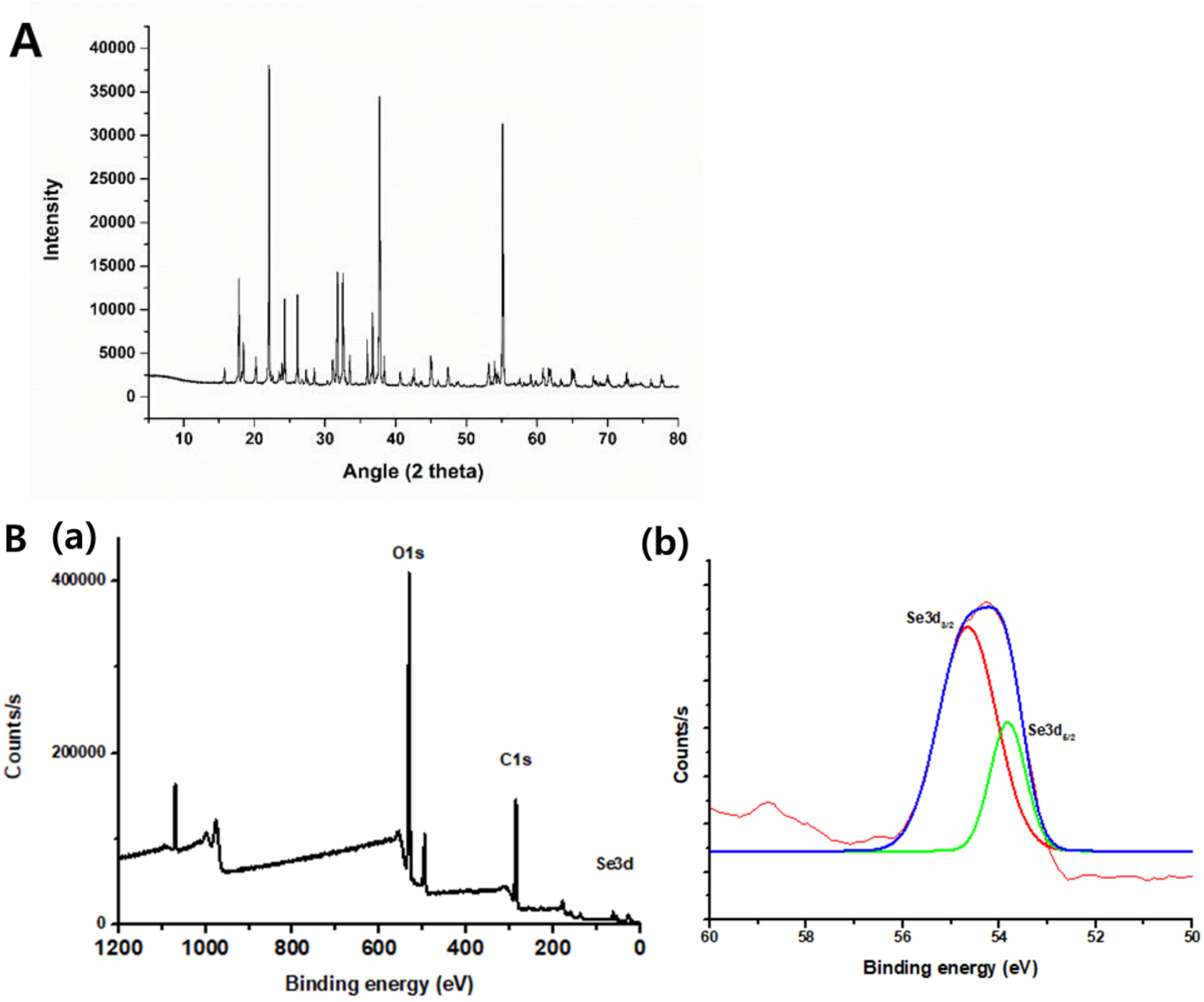

Supplementary Figure 3. Physicochemical characterization. (A) XRD image of sodium selenite. (B) Full XPS scan (a) and narrow XPS scan (b) of SeNPs.

© 2025. Sharma G et al. Published online at http://www.aginganddisease.org/EN/10.14336/AD.2025.0025

SUPPLEMENTARY DATA

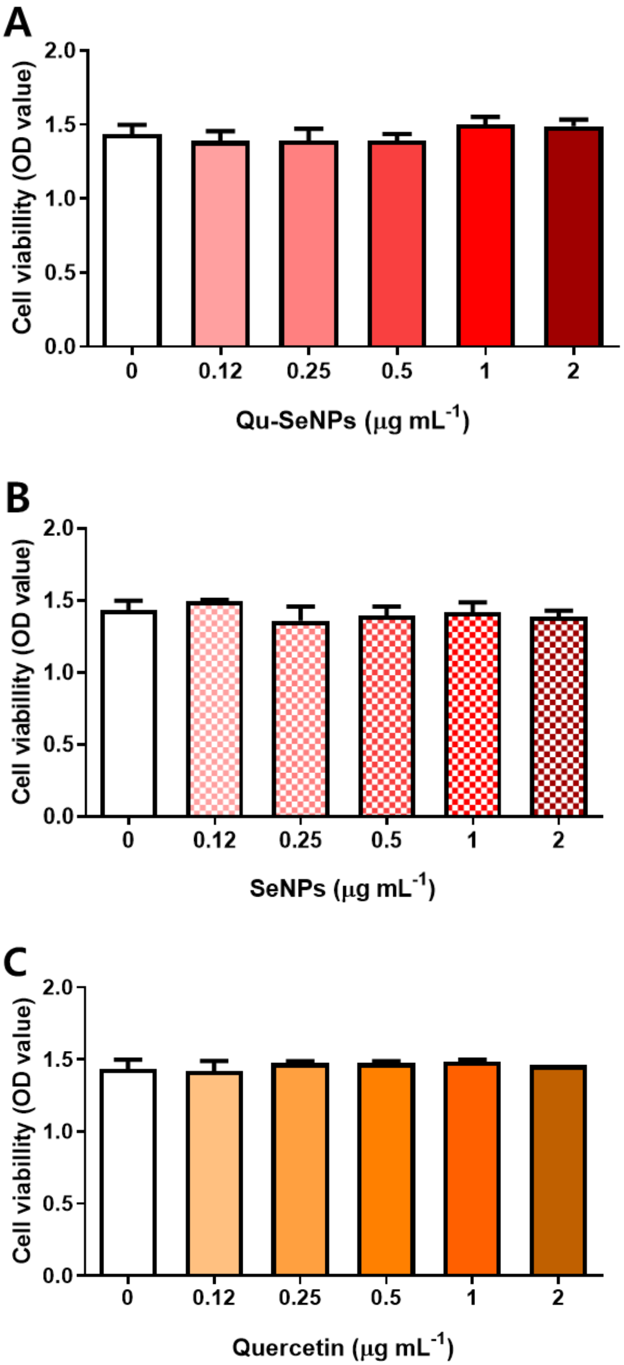

Supplementary Figure 4. Cell cytotoxicity assays for QuSeNPs, SeNPs, and Qu.

© 2025. Sharma G et al. Published online at http://www.aginganddisease.org/EN/10.14336/AD.2025.0025

SUPPLEMENTARY DATA

Supplementary Table 1. Mouse primers for real-time RT-PCR.

Gene	Primer sequence (5´-3´)
Osterix	F: GGAAAGGAGGCACAAAGAAGCCAT<br>R: AGTCCATTGGTGCTTGAGAAGGGA
Col1a	F: TTCTCCTGGCAAAGACGGAC<br>R: AGGAAGCTGAAGTCATAACCGCCA
Osteocalcin	F: TGCTTGTGACGAGCTATCAG<br>R: GAGGACAGGGAGGATCAAGT
Osteopontin	F: CCACAATGAACAAGTGGCTGTGCT<br>R: TAGGTAGGTGCCAGGAGCACATTT
Runx2	F: AAGTGCGGTGCAAACTTTCT<br>R: TCTCGGTGGCTGGTAGTGA
Bone sialoprotein	F: TCCCAGGTGTGTCATTGAAGA<br>R: GGTATGTTTGCGCAGTTAGCAA
RANKL	F: CGTGCAGAAGGAACTGCAACACAT<br>R: TAGGTAGGTGAGGTGTGCAAA
Connexin 43	F: CGCAAGGATGACACGCAAAT<br>R: ATTTGCGTGTCATCCTTGCG
Cathepsin K	F: AGGCAGCTAAATGCAGAGGGTACA<br>R:ACCTTGCATCGATGGACACAGAGA
NFATc1	F: GGTGCCTTTTGCGAGCAGTATC<br>R: CGTATGGACCAGAATGTGACGG
Osteoprotegerin	F:CCACAATGAACAAGTGGCTGTGCT<br>R:TAGGTAGGTGCCAGGAGCAC
U6	F: CTCGCTTCGGCAGCACATATACT<br>R: ACGCTTCACGAATTTGCGTGTC
miR-206	F: GGGTGGAATGTAAGGAAGT<br>R: CGTGTCGTGGAGTC
GAPDH	F: TCGTGGATCTGACGTGCCGCCTG<br>R: CACCACCCTGTTGCTGTAGCCGTAT

© 2025. Sharma G et al. Published online at http://www.aginganddisease.org/EN/10.14336/AD.2025.0025
